# ACT-PRESTO: Rapid and consistent tissue clearing and labeling method for 3-dimensional (3D) imaging

**DOI:** 10.1038/srep18631

**Published:** 2016-01-11

**Authors:** Eunsoo Lee, Jungyoon Choi, Youhwa Jo, Joo Yeon Kim, Yu Jin Jang, Hye Myeong Lee, So Yeun Kim, Ho-Jae Lee, Keunchang Cho, Neoncheol Jung, Eun Mi Hur, Sung Jin Jeong, Cheil Moon, Youngshik Choe, Im Joo Rhyu, Hyun Kim, Woong Sun

**Affiliations:** 1 Department of Anatomy and Division of Brain Korea 21 Plus Program for Biomedical Science, Korea University College of Medicine, Anam-dong, Seongbuk-gu, Seoul 136-705, Korea; 2 Department of Neural Development and Disease, Korea Brain Research Institute, 701-300 Daegu, Korea; 3 Department of Brain & Cognitive Sciences, Graduate School, Daegu Gyeungbuk Institute of Science and Technology (DGIST), Daegu, Korea; 4 Logos Biosystems, Inc. Anyang-Si, Gyunggi-Do, 431-755, Republic of Korea; 5 Center for Neuroscience, Brain Science Institute, Korea Institute of Science and Technology, Seoul, Korea; 6 Department of Neuroscience, Korea University of Science and Technology (UST), Daejeon, Korea

## Abstract

Understanding the structural organization of organs and organisms at the cellular level is a fundamental challenge in biology. This task has been approached by reconstructing three-dimensional structure from images taken from serially sectioned tissues, which is not only labor-intensive and time-consuming but also error-prone. Recent advances in tissue clearing techniques allow visualization of cellular structures and neural networks inside of unsectioned whole tissues or the entire body. However, currently available protocols require long process times. Here, we present the rapid and highly reproducible ACT-PRESTO (*a*ctive *c*larity *t*echnique-*p*ressure *r*elated *e*fficient and *s*table *t*ransfer of macromolecules into *o*rgans) method that clears tissues or the whole body within 1 day while preserving tissue architecture and protein-based signals derived from endogenous fluorescent proteins. Moreover, ACT-PRESTO is compatible with conventional immunolabeling methods and expedites antibody penetration into thick specimens by applying pressure. The speed and consistency of this method will allow high-content mapping and analysis of normal and pathological features in intact organs and bodies.

Volume imaging with single-cell resolution should allow molecular and structural analyses of biological systems^1^,^2^,^3^ and enable more accurate medical diagnosis^4^,^5^. Conventional volume imaging requires tissue sectioning, labeling of serially sectioned tissues with probes for specific targets, such as macromolecules (proteins and nucleotides), and reconstructing individual two-dimensional (2D) images into three-dimensional (3D) structures^6^,^7^,^8^,^9^,^10^,^11^,^12^. Such processes are not only labor-intensive and time-consuming, but also prone to errors, such as mechanical distortion of tissues caused by sectioning and inaccurate 3D reconstruction due to the complexity of mapping reference points.

Development of methods to transform tissues and organs into optically transparent samples and image cellular structures in unsectioned, intact organs has attracted attention^1^,^2^,^3^,^7^,^13^,^14^,^15^,^16^,^17^,^18^,^19^. One way to achieve transparency is immersing samples into selective media with a suitable refractive index (approximately 1.45) to minimize light scattering^20^,^21^. Several hydrophobic reagents have been developed to render tissues nearly transparent, but many of these reagents cause rapid quenching of fluorescent signals during the dehydration step^19^,^22^. Hydrophilic reagents have been introduced to maintain fluorescent signals^1^,^13^,^15^, but optical clearing is generally slow, particularly for large tissues.

Substantial progress has been made in tissue (brain in particular) clearing and promoting the penetration of labeling reagents with the advent of hydrogen-based clearing methods^2^,^3^,^23^. These methods include a protein and acrylamide crosslinking step that selectively immobilizes proteins and other macromolecules, resulting in a tissue-embedded hydrogel^24^,^25^. Lipid components are selectively removed either by passive diffusion or actively by electrophoresis. Removing the lipid components markedly increases optical transparency, and the porous nature of the hydrogel allows penetration of labeling reagents deep inside thick tissues^2^,^3^. However, hydrogel-embedded tissue clearing methods retain a dense extracellular matrix (ECM), which hinders penetration of macromolecules into thick organs. Although recent advances in tissue clearing and labeling techniques have greatly improved methods to visualize molecules deep inside organs, the entire process is slow and requires complex procedures. For example, clearing the entire mouse brain with CLARITY takes at least 1–2 weeks and requires a specialized device. Other methods, such as CUBIC (*c*lear, *u*nobstructed *b*rain *i*maging *c*ocktails and *c*omputational analysis) and iDISCO (*i*mmunolabeling-enabled 3*D i*maging of *s*olvent-*c*leared *o*rgans), do not require specific equipment and are scalable, but these methods rely on free diffusion of buffers, which delays processing time and makes complete clearing difficult^1^,^3^,^16^. PACT (*pa*ssive *c*larity *t*echnique) takes advantage of a tissue hydrogel in a porous clearing system. Although systemic perfusion of the clearing solution substantially reduces clearing time (weeks for whole mouse clearing), passive diffusion clearing is still slow, preventing its application for large volume tissues or whole-organism clearing^2^. Therefore, a significant challenge in this field is to develop an improved methodology to deliver reagents deep inside of thick tissues to achieve whole-tissue and body imaging with single-cell resolution in a reasonable time.

In this study, we report the rapid, efficient, and scalable ACT (*a*ctive *c*larity *t*echnique) method, which renders large tissue samples optically transparent and enables labeling of deep structures. We show that ACT allows clearing of whole organs within 4–20 hours, which is partly possible by optimizing formation of the tissue-hydrogel and applying electrical current. Furthermore, this method is scalable for large organs (e.g., rat or rabbit brains) or even whole bodies of adult animals (e.g., adult mice, zebrafish, and *Xenopus*). We also demonstrate that PRESTO (*p*ressure *r*elated *e*fficient and *s*table *t*ransfer of macromolecules into *o*rgans) facilitates active penetration of macromolecules, such as antibodies, deep into dense organs by applying centrifugal pressure or convection flow to the organs. The rapid nature, compatibility with immunolabeling, and scalability of ACT should expedite volume imaging and medical diagnosis in 3D.

## Results

### ACT using a dense current electrophoretic tissue clearing (ETC) system

**Figure 1:**
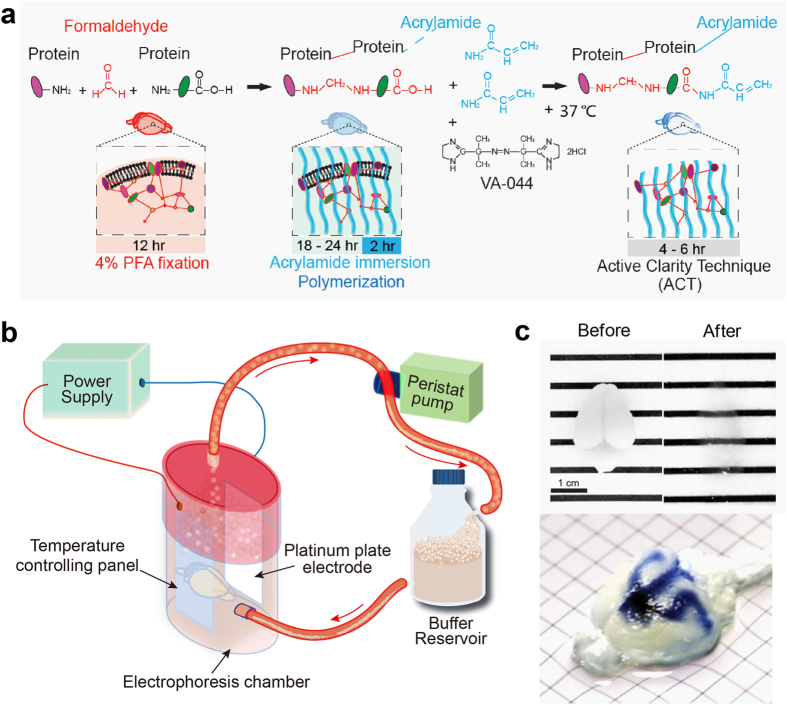
Active Clarity Technique (ACT) is a rapid and efficient whole-brain clearing technique. (**a**) Workflow for clearing tissue with the ACT. The brain was isolated from a cardiac-perfused animal and post-fixed in 4% paraformaldehyde (PFA) overnight to induce DNA-protein crosslinking. The brain was immersed in a 4% acrylamide and thermal initiator-containing solution for 18–24 hours. The whole brain was incubated at 37 °C for 2 hours. After polymerization, lipid membranes were removed by ACT for 4–6 hours. (**b**) Diagram of the ACT-ECT system. (**c**) Brains before (*left*) and after (*right*) ACT processing; C57BL/6 mouse brains after polymerization, ACT, and refractive index matching. Scale bar, 1 cm. Injection of trypan blue into the ventricle of a cleared brain (*bottom*). Square unit; x: 5 mm, y: 5 mm.

We started by optimizing previously reported CLARITY tissue clearing processes by modifying conditions for tissue-hydrogel polymerization and electrophoresis (Fig. 1a). Because tissue clearing can be affected by the fixation procedure, we employed a two-step fixation protocol as reported previously for passive tissue clearing^2^: paraformaldehyde (PFA) fixation followed by acrylamide infusion without bis-acrylamide, prior to ACT. Clearing using ACT results in less protein-acrylamide crosslinking, compared to that of CLARITY, resulting in a higher porosity hydrogel to allows rapid extraction of lipids and better diffusion of macromolecules^2^. The original CLARITY protocol includes an electrophoresis step to actively remove lipids, which has raised concerns with regard to consistency^23^. Multiple factors have been suggested to cause inconsistencies, including the unstable temperature, rapid changes in pH, and blocking of current by bubbles.

**Figure 2:**
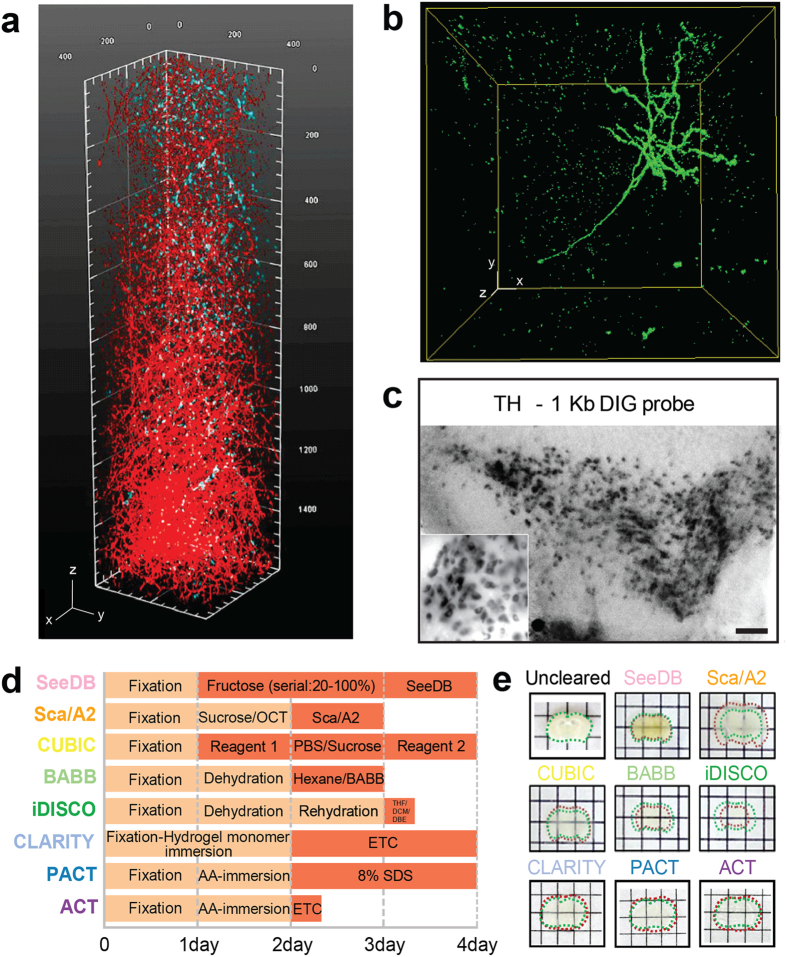
Active Clarity Technique (ACT) for three-dimensional imaging of protein and RNA distribution in adult brain samples. (**a**) Unsectioned mouse brain tissue image showing part of the midbrain stained with tyrosine-hydroxylase (20 × objective; stack size, 1,580 μm; step size, 2 μm). Scale bar, 100 μm. (**b**) Neurons labeled with the adeno-associated virus-green fluorescent protein (AAV-GFP) and immunolabeling of dendritic arbors with anti-GFP antibodies (10 × objective, 0.7 × confocal zoom; stack size, 1,180 μm; step size, 2 μm). Scale bar, 100 μm. (**c**) *In situ* hybridization of ACT-processed midbrain slice with 1.1 Kb DIG-labeled tyrosine hydroxylase (TH) probes. Scale bar, 100 μm. (**d**) Comparison of procedures and processing times between ACT and other clearing methods. (**e**) Images of brain blocks (1-mm thick) after processing. Dotted green lines indicate original sizes of blocks and red lines mark sizes after clearing. Square unit; x: 5 mm, y: 5 mm.

Thus, we designed a modified version of the ETC chamber system using a platinum plate to generate a dense regular current in the ETC chamber. Therefore, the quality of clearing solution was maintained without changing pH or color during the extended ETC period (Supplementary Fig. 1). Moreover, adding an active cooling system greatly reduced heat generation. In addition, we designed a long ETC chamber, which allowed all air bubbles to float to the top where they were removed through the top outlet of the chamber (Fig. 1b and Supplementary Fig. 1). Therefore, we could rapidly obtain cleared tissue without tissue surface burning, collapse, or protein-loss. We found that 6 hours of ETC transformed brain tissue to nearly completely transparent while preserving protein content (Fig. 1c, Supplementary Figs 2a and 3a, Supplementary Video 1). Injecting trypan blue into the ventricle of the cleared brain revealed the architecture of the lateral ventricle of the whole brain (Fig. 1c). The organization of the immunolabeled dopaminergic neurons in the midbrain (Fig. 2a) and motoneurons in the entire spinal cord (Supplementary Fig. 2b and Supplementary Video 2) became apparent by imaging with a selective plane illumination microscope. When a mouse brain that had been injected with adeno-associated virus-green fluorescent protein (AAV-GFP) was cleared with ACT and imaged (Supplementary Fig. 3b), GFP fluorescence signals and GFP antibody-immunolabeled signals overlapped. Furthermore, dendritic arborization of a deep-layer neuron was clearly visible (Fig. 2b, Supplementary Video 3). ETC can be destructive and cause loss of tissue integrity. However, we found that clearing for 12 hours did not significantly reduce the integrity of the tissue-hydrogel, and tissues that swelled during initial clearing recovered to their original size in the refractive index matching solution (RIMS). (Supplementary Fig. 3c). Protein content was not reduced in the tissue-hydrogel during 12 hours of ETC (Supplementary Fig. 3d). We confirmed that the glial fibrillary acidic protein (GFAP) and neuronal nuclei (NeuN) signals, which label astrocytes and neurons, respectively, were readily detectable after 12 hours of ETC (Supplementary Fig. 3e). We have tested 82 commercially available antibodies that recognize various antigens in membranes, the cytoplasm, nuclei, and the ECM (Supplementary Fig. 4 and Supplementary Table 2), and found that 91.5% of the antibodies (75 antibodies) labeled the target structures in thick specimens, suggesting that the ACT protocol is compatible with standard immunolabeling procedures and widely used antibodies. Small but significant loss of immunoreactivity against some antibodies may be caused by the masking of specific epitopes by the formation of tissue-hydrogel conjugates. In addition, we confirmed that 1-Kb DIG-labeled RNA probes readily penetrated the sample and labeled tyrosine-hydroxylase (TH) neurons (Fig. 2c), demonstrating that the ACT-processed tissue-hydrogel can be adopted for detecting many different macromolecules, including proteins and nucleic acids.

To compare the efficacy of ACT with other clearing methods, we selected seven methods (SeeDB, Sca*l*eA2, CUBIC, BABB, iDISCO, CLARITY, and PACT) compatible with immunolabeling protocols. Two hours of ACT clearing was sufficient to achieve nearly complete optical transparency in 1 mm brain sections. However, the other methods required 1–3 days to achieve optical transparency comparable to that achieved by ACT (Fig. 2d, Supplementary Fig. 2c). Samples became nearly completely transparent after processing using organic solvent-based methods, such as BABB and iDISCO, whereas samples processed by other methods that preserve lipids, such as SeeDB and Sca*l*e, were less transparent even after adjusting the refractive index (RI). The extent of optical transparency achieved by the CLARITY-based methods (CLARITY, PACT, and ACT) was comparable to that achieved by organic solvent-based methods. CLARITY and PACT procedures are known to cause transient swelling of samples during exposure to SDS-containing de-lipidation buffer. Similarly, samples processed by ACT expanded about 80% in size (Supplementary Fig. 3c). However, samples returned to their original size in RIMS (Supplementary Figs 2d and 3c). In contrast to acrylamide-based methods, organic solvent-based methods markedly shrank tissues to approximately half of their original size, which could prevent a precise analysis of cellular or tissue architecture. Changes in volume were less dramatic after processing by SeeDB and CUBIC (Fig. 2e). Collectively, these results show that the ACT method facilitates clearing process with recovery to original size (Fig. 2e and Supplementary Fig. 2d).

### Scalability of ACT

**Figure 3:**
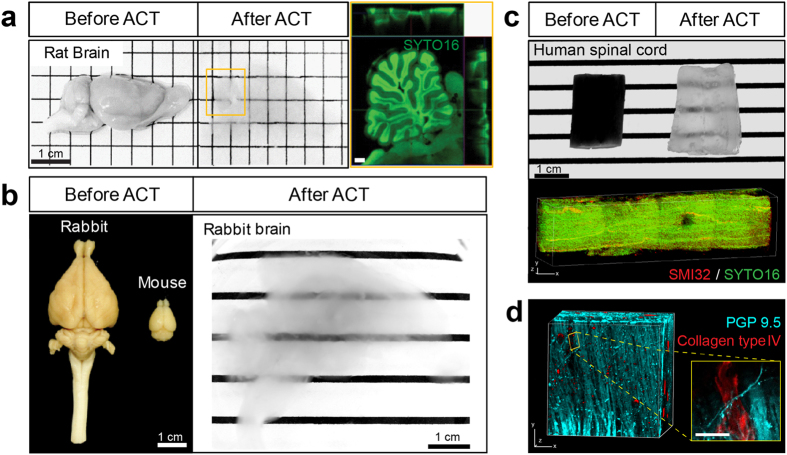
Scalability of the Active Clarity Technique (ACT). (**a**) Rat brain hemisphere processed with 15 hours of ACT. Scale bar, 1 cm. (*Right*) Magnified fluorescent image of the boxed region in the middle (yellow). Nuclear-staining (SYTO16) of rat cerebellum (4 × 4 tile scan with the Fluar 5 × objective; stack size, 2,580 μm; stack step, 20 μm) acquired with the LSM 700 microscope. Scale bar, 1 mm. (**b**) Size comparison of an adult mouse brain and an adult rabbit brain (*left*). The rabbit brain was cleared after 36 hours of ACT (*right*). (**c, d**) Human spinal cord block cleared with ACT and immunolabeled. (**c**) Before and after ACT. Scale bar, 1 cm. Human spinal cord block (1.3–1.5 cm thick) was cleared (100 hours of ACT) and stained with SMI32 (red). SYTO16 (green) was used for nuclear staining (1.1 × confocal zoom; stack size, 1,020 μm; stack step, 1 μm). (**d**) Spinal cord block was immunostained for PGP 9.5 (cyan) and collagen type IV (red) (2 × confocal zoom; stack size, 420 μm; stack step, 5 μm). All three-dimensional reconstructed images were obtained with a Zeiss 780 (**c**) or 700 (**d**) confocal microscope with a Plan-apochromat 10 ×/0.45 M27 lens. Scale bar, 50 μm.

Because ACT enables rapid clearing of tissues, we tested whether it could be used to clear larger brains within a reasonable time. For an adult rat brain (about 2 g, within x: 1.6 mm, y: 3.3 mm, and z: 1.3 mm), which is approximately four-times larger than an adult mouse brain (about 0.5 g, within x: 1.0 mm, y: 1.6 mm, and z: 0.8 mm), 15 hours of ETC was sufficient to clear the entire brain (Fig. 3a), and we could observe the entire architecture of the cerebellum after nuclear staining. For rabbit brain (about 10 g, within x: 3.6 mm, y: 5.5 mm, and z: 2.9 mm), which is about 45-times larger (volume) than a mouse brain, 50 hours of ACT was sufficient to render the hemi-sectioned brain nearly completely transparent, but with small deformations due to physical constraints of the ETC chamber, which was designed for a mouse brain (Fig. 3b). We also cleared human spinal cord tissue fixed with formalin. A spinal cord (1.3 cm diameter) was dissected out of a cadaver, followed by 100 hours of ACT clearing (Fig. 3c). Double-labeling with protein gene product 9.5 and collagen type IV clearly revealed the spinal cord architecture (Fig. 3d). These results show that ACT is scalable to large tissues by longer processing. We found a rough linear relationship between time to clear and tissue thickness, with a clearing speed of 0.5–1 mm/hour. However, it should be noted that clearing is affected by many other factors, such as contents of lipids and connective tissues and fixation conditions; thus, we recommend to determine optimal clearing conditions empirically for a particular tissue.

### Peripheral organ clearing with ACT

**Figure 4:**
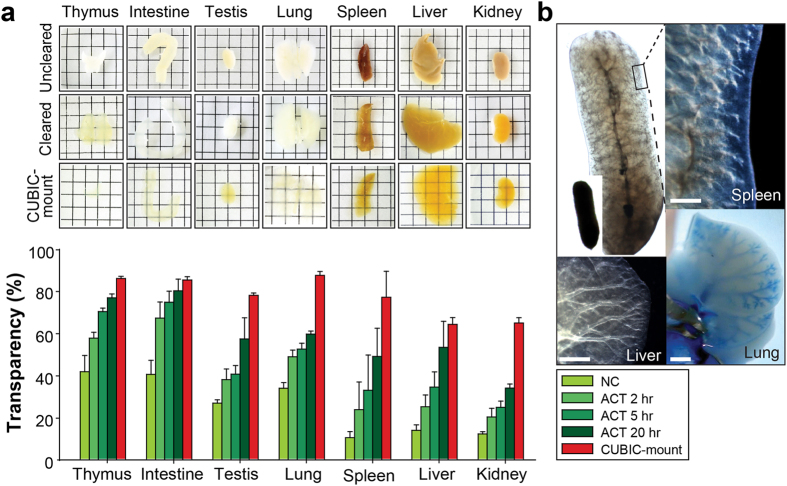
Active Clarity Technique (ACT) for whole-organ clearing and immunolabeling. (**a**) Cleared organ samples (thymus, intestine, testis, lung, spleen, liver, and kidney) were treated with CUBIC-mount for 5 days to adjust the refractive indices of the tissue and reagent. Square units; x: 5 mm, y: 5 mm. (**b**) Transmission images of cleared whole organs (liver, lung, kidney, and spleen) by ACT. Images were taken after electrophoretic tissue clearing (ETC) in PBS. Details of the tissue architecture were visualized with a standard dissection microscope. Images were acquired on an Olympus BX53 digital microscope DP73 camera. Scale bar, 1 mm.

We applied the ACT protocol to other peripheral organs (Fig. 4a). We selected seven tissues (thymus, intestine, testis, lung, spleen, liver, and kidney), which were cleared overnight with ACT in 20 hours. Relatively thin and white-colored tissues (e.g., thymus and intestine) were completely transparent following ETC, whereas thick or colored organs remained opaque after overnight clearing (Supplementary Fig. 5a). Lipid removal by ETC was insufficient to achieve complete optical transparency of thick connective tissues with a rich ECM. Immersing such organs in the RIMS solution markedly improved clearing (Supplementary Fig. 5c). Recently, it has been reported that a compound in the CUBIC cocktail, aminoalcohol leads to tissue decolorization by releasing heme from blood within samples^1^,^18^. We developed a recipe to adjust RIs by modifying the CUBIC solution, and called this mounting solution CUBIC-mount, which included sucrose, urea, and N,N,N′,N′-tetrakis(2-hydroxypropyl)ethylenediamine. CUBIC-mount had RI values of 1.43–1.48 and rendered tissues transparent (Fig. 4a and Supplementary Fig. 5b). Immersing brown tissues for longer (5 days) markedly increased transparency, and the optimal time for organ clearing was empirically determined (Supplementary Fig. 5b and Supplementary Table 4). CUBIC-mount provides a cost-effective way to adjust RIs compared with other RI-matching solutions including RIMS, sRIMS^2^, and TDE^26^ (Supplementary Fig. 5c). The estimated cost to produce CUBIC-mount and RIMS is $12 and $80–85/10 ml solution, respectively.

Of note, ACT in PBS provided high-contrast between connective and soft tissues, and tissue architecture became clearly visible even under a standard dissection microscope. These results show that ACT processing facilitates the identification and analysis of structures in large organs at a macroscopic level (Fig. 4b).

### PRESTO for rapid antibody penetration in dense tissues

**Figure 5:**
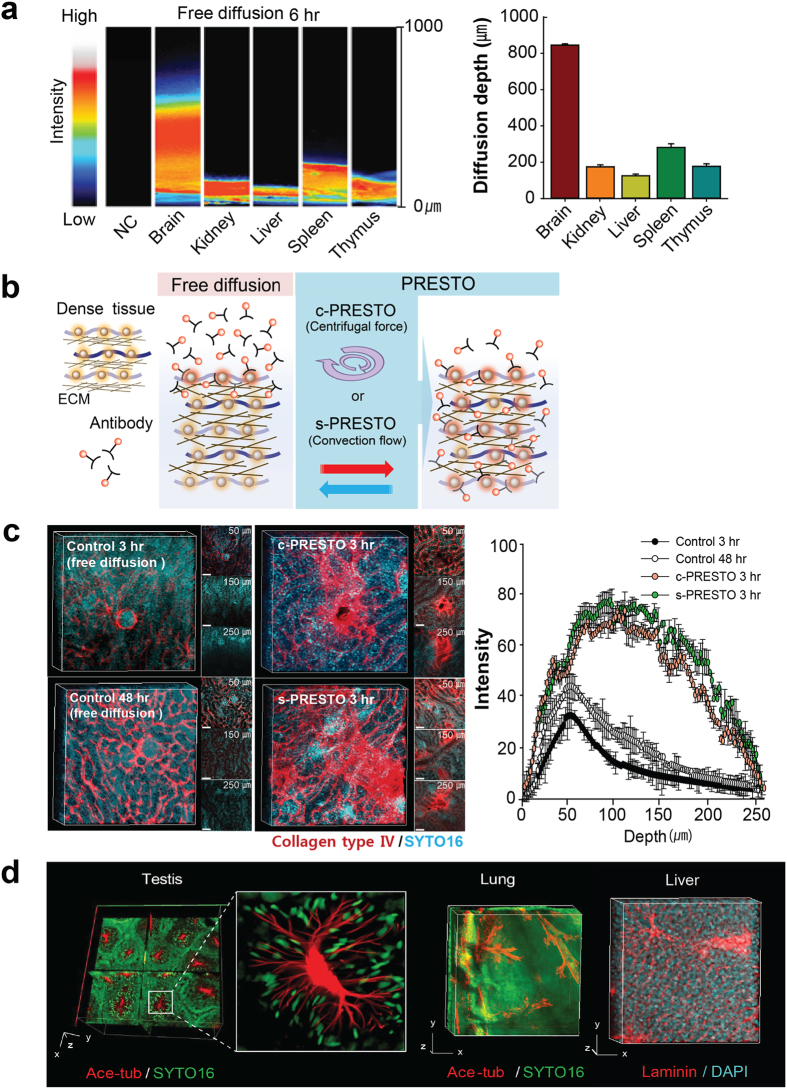
ACT-PRESTO (*a*ctive *c*larity *t*echnique-*p*ressure *r*elated *e*fficient and *s*table *t*ransfer of macromolecules into *o*rgans) for rapid immunolabeling of dense tissues. (**a**) Comparison of diffusion rate using ACT-processed organs. (**b**) Schematic diagram for dense tissue immunohistochemistry. Tissues for *c*entrifugal PRESTO (c-PRESTO) were centrifuged at 600 × g for 3 hours using a standard table-top centrifuge to expedite penetration of the primary and secondary antibodies. A syringe pump was used for the antibody reaction during *s*yringe PRESTO (s-PRESTO). (**c**) Kidneys were labeled with collagen type IV using various protocols. Note that 3 hours of c- or s-PRESTO markedly enhanced the depth of specific labeling compared to that of the controls. Three-dimensional (3D) reconstructed images were obtained with a Zeiss 700 confocal microscope with a Plan-apochromat 10 ×/0.45 M27 lens, 2 × confocal zoom (stack size, 200 μm; stack step, 2 μm), and post-processed with Vaa3D software. Scale bar, 100 μm. Depth of fluorescence intensity was greater in PRESTO-treated tissue compared to that of free-diffusion labeled samples using ACT processed kidney tissue (mean ± standard deviation, n = 5). (**d**) Reconstituted 3D images of testis, lung, and liver. The organs were stained with acetylated tubulin (red in testis and lung) or laminin antibodies (red in liver). SYTO16 or DAPI were used for nuclear staining of the organs. Images were obtained with a Zeiss 700 confocal microscope with a Plan-apochromat 10 ×/0.45 M27 lens, 2 × confocal zoom (testis; stack size, 600 μm; stack step, 5 μm; liver; stack size, 226 μm; stack step, 2 μm; Scale bar, 100 μm), with a EC Plan-Neoflua 5 ×/0.16 M27 lens (lung; stack size, 1,265 μm; stack step, 5 μm; Scale bar, 500 μm).

Protein-enriched connective tissues remained after ACT, particularly those in dense organs. The ECM is a physical barrier for penetration of antibodies into tissues^27^,^28^; thus, antibody penetration into peripheral organs was much slower than that into soft organs, such as brain (Fig. 5a). To expedite antibody diffusion, we designed a method to actively infuse macromolecules and deliver reagents into dense structures and called it PRESTO (*p*ressure *r*elated *e*fficient and *s*table *t*ransfer of macromolecules into *o*rgans) (Fig. 5b). A 3 hour incubation of kidney tissue with antibodies labeled structures 10–30 μm deep (Fig. 5c,d) and applying centrifugal force using a standard table-top centrifuge (600 rcf) (*c*entrifugal PRESTO [c-PRESTO]) markedly facilitated antibody delivery (Fig. 5c,d). When we applied the c-PRESTO procedure to normal tissues, 3 hours was sufficient to label structures 120 μm deep, which was deeper than 2 day-labeling by shaking (Fig. 5c,d). We also designed a protocol to apply convection flow^29^,^30^ with a syringe pump (*s*yringe PRESTO [s-PRESTO]). The pump infuses a solution containing the labeling reagents into the specimen. The pumping direction changes when the designated volume is reached and withdraws the solution after a certain period. s-PRESTO infusion improved antibody penetration, and we could image fluorescent proteins at a depth four times greater than that in control tissue (Fig. 5c). These results clearly show that c-PRESTO and s-PRESTO allow easy volume imaging of various organs, including testis, kidney, and lung (Fig. 5d).

### ACT is useful for whole-body clearing of various organisms

**Figure 6:**
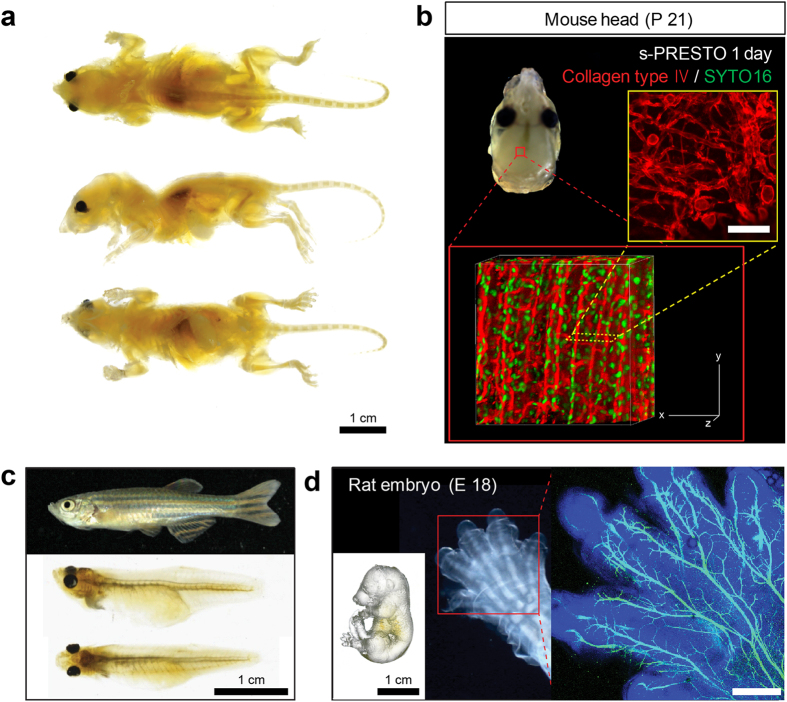
Whole body clearing of various organisms using the Active Clarity Technique (ACT). (**a**) Whole mouse body (3-weeks-old) was cleared in 24 hours using the ACT. After 3 days of incubation in CUBIC-mount solution, the ACT-processed mouse whole body became optically transparent and could be imaged without sectioning. Scale bar, 1 cm. (**b**) Image shows collagen type IV-labeled extracellular matrix (ECM) within the skull and underlying meninges. Images were obtained with a Zeiss 700 confocal microscope with a Plan-apochromat 10 ×/0.45 M27 lens, 2 × confocal zoom (stack size, 320 μm; stack step, 5 μm). Scale bar, 100 μm. (**c**) Comparison of optical transparency of whole zebrafish body before and after ACT clearing. Scale bar, 1 cm. (**d**) Whole rat embryo body (embryonic day 18) was cleared by the ACT. Scale bar, 1 cm. Embryo labeled with TrkA antibody shows the details of TrkA innervation in the foot. Imaged obtained with a Zeiss 700 confocal microscope with a Plan-Neofluar 5 ×/0.15 objective, 0.7 × confocal zoom (maximum projection; stack size, 1,250 μm; stack step, 15 μm). Scale bar, 500 μm.

Whole-body clearing can be achieved by modifying the CLARITY and CUBIC protocols. However, these methods require >2 weeks, due to passive solution exchange^1^,^2^,^18^. The short processing time of ACT makes this method applicable to much larger specimens, such as the whole body (Fig. 6). To this end, we removed skin from young mice (3-weeks-old and weight, 25 g) and placed the animal in the chamber for ACT processing. Whole-body clearing was achieved within 24 hours (Fig. 6a) and dissection of the organs confirmed that most were optically transparent (Supplementary Fig. 6a). We occasionally found that the entire organism turned yellowish, presumably due to the Mailard reaction^13^ or an effect of endogenous chromophores inside the tissues^18^. Preventing the Mailard reaction with alpha-thioglycerol might improve transparency. However, the brain inside the skull remained partially opaque, indicating that the skull hindered but did not block the clearing process. After ACT processing, we visualized the meningeal distribution of blood vessels after collagen immunolabeling (Fig. 6b). Substantial transparency was achieved following ACT of other organisms, such as zebrafish (Fig. 6c), rat embryos (Fig. 6d), chick embryos, *Xenopus*, and small octopus (Supplementary Fig. 6b).

## Discussion

Optical clearing techniques compatible with standard immunolabeling methods will revolutionize the way molecular and cellular details of organs and organisms are studied. Two of the technical limitations of currently available methods are long processing time for clearing and inefficient antibody labeling. In this study, we developed a method that (i) expedites the clearing and labeling steps while preserving cellular and subcellular structures and (ii) is compatible with standard probes to detect proteins and nucleic acids. The original CLARITY protocol requires several weeks for a whole mouse brain to clear and label with specific proteins, and the modified protocol with passive clearing, such as PACT, requires months. Although use of organic or inorganic solvents with the desired RI (RI ~1.5) may reduce time for clearing and labeling whole-mouse embryos (E16) to weeks, endogenous fluorescent signals disappear during the clearing procedure, and these methods are incompatible with post-immunostaining. We showed that ACT tissue clearing can be completed within hours to days and that the entire process of 3D immunohistochemistry from sampling to image acquisition can be completed within 1 week.

Procedures that primarily preserve lipid components (SeeDB and Sca*l*eA)^13^,^15^ are relatively simple, but tissues are only partially cleared in most cases. Furthermore, the rate of macromolecule penetration to deliver proteins and nucleic acid probes is slow, which limits such methods to clearing small and/or soft tissues. Sca*l*eS is a recently reported tissue clearing technique^31^ that improves tissue preservation for immunochemical labeling and maintains the original tissue structure. However, Sca*l*eS also has long tissue processing time, making it insufficient for larger-scale connectome studies. Organic solvent-based methods (iDISCO and BABB)^7^,^16^, which remove lipid components, provide a relatively simple way to aid antibody penetration for volume imaging. However, organic solvent-based methods cause significant shrinkage of specimens, which precludes detailed a quantitative morphological analysis. Transgenically expressed fluorescent proteins lose their fluorescence during clearing and must be detected by immunolabeling, making it difficult to distinguish widely used GFP variants, such as cyan and yellow fluorescent proteins. Furthermore, the organic solutions used in these procedures are toxic, and special care must be exercised.

CUBIC clearing includes a hydrophilic solvent-based lipid elimination procedure and adjustment of RIs. The CUBIC procedure is relatively simple, the changes in tissue size are marginal, and processed samples become highly transparent. It also maintains the fluorescence of GFP variants and is compatible with immunolabeling. However, the main weakness of passive clearing methods is the lengthy processing time and lipid-rich regions remains opaque after processing. Notably, our large-scale analysis of antibody labeling demonstrated that the ACT and CUBIC methods have different compatibility, indicating that the two protocols can be selected depending on the epitopes of interest (Supplementary Fig. 7). The CUBIC and CLARITY methods can be combined^1^,^3^ and immersion of the ACT-processed tissue-hydrogel into CUBIC-mount rapidly adjusted the RIs and rendered the samples optically transparent, similar to RIMS or FocusClear. As CUBIC reagents are much cheaper than RIMS or FocusClear reagents, they may be more widely used, particularly for clearing large specimens. Here, we have formulated a mounting cocktail (CUBIC-mount), which includes aminoalcohols for bleaching and sucrose and urea for adjusting the RI. Fluoropores are stable in CUBIC-mount, and loss of fluorescently labeled signals was not evident for weeks during our preliminary observations.

We optimized ETC conditions for efficient and consistent tissue clearing and found that thin platinum wire electrodes often generated heat during electrolysis, which turned the ETC buffer brown and burned tissues. Use of a wide platinum plate and adding an active cooling system markedly prevented heat. In addition, excessive bubbles generated during circulation of the buffer perturbed the ionic currents and delayed the ETC process. To circumvent this problem, we designed a long ETC chamber so air bubbles could float to the top and were removed by the outlet located at the top of the chamber.

Tissue clearing is affected by cell density as well as the extent and composition of lipid and ECM components. In general, hard tissues, such as kidney and liver, required longer time to clear compared to that of soft tissues (Supplementary Table 4). Most adult mouse tissues were cleared after 2–20 hours of ETC, but protein-enriched ECM fibers remained opaque after ACT processing. Clearing time/efficacy and the extent of changes in tissue size differ among different tissues when clearing a whole body and considerable swelling occurred in most organs under our regular conditions. Therefore, caution should be exercised when clearing organs with physical constraints arising from several features, such as high ECM contents (e.g., liver and kidney) or encapsulation by a tough sac (e.g., testis) or bones (e.g., brain and spinal cord). In particular, clearing occasionally resulted in hydrocephalus-like rupture of the embryonic head. In this case, increasing the ionic concentration of the buffer (≥0.01 M PBS) prevented rupture of samples and enabled desired clearing and subsequent labeling.

Animals, such as zebrafish and *Xenopus*, are widely used as model organisms in developmental biology, as their embryos are transparent. However, they gain color as they mature, limiting the examination of anatomical structures or molecules of interest in adult animals. As ACT processing clears adult zebrafish and *Xenopus*, ACT will not only permit examination of molecules, structures, and cellular networks in intact adult organs but will also enable investigating changes in such features during development into an adult. Of note, melanin pigments in the adult skin of these animals remained even after clearing. In contrast to lipid-soluble heme, which is removed during clearing, melanin is conjugated to a protein globulin^32^,^33^; thus, it cannot be removed by our protocol. Therefore, if complete removal of color is desired, chemical bleaching by other methods, such as oxalic acid^34^ or H_2_O_2_^35^, should be used for melanin-rich tissues, such as the retina and skin.

Organ or whole-body clearing enables fine-scale subcellular analysis of cleared specimens (Fig. 6b), but ACT clearing also provides a powerful method for system-level identification and analysis of tissue structure and the cellular networks in intact specimens. Luminal structures were well maintained after ACT clearing, and injecting dye revealed the luminal structures, such as ventricular systems in the brain and pulmonary branches. Furthermore, the fine structures of the liver and spleen secretory ducts were easily detected under a standard dissection microscope, and the internal structures of organs and bones were easily identified following whole-body clearing.

One of the benefits of forming a tissue-embedded hydrogel is that the highly porous nature of the hydrogel expedites penetration of macromolecular-labeling reagents. Removing lipids in lipid-rich structures, such as the brain, produces large pores. As pores form after tissue-hydrogel formation, removing the lipid does not cause loss of protein. However, removing lipid alone is insufficient to render optical transparency in dense organs with high ECM content, and the high ECM density hinders penetration of macromolecules. Here, we showed that applying centrifugal pressure or convection flow significantly promoted penetration of macromolecules into deep structures. Similarly, PEA-CLARITY, a recently reported technique for clearing plants, uses negative pressure on plant samples^36^, suggesting that positive or negative pressure can be used to deliver macromolecules into tissues from diverse organisms.

Another advantage of the PRESTO technique is its compatibility with standard immunolabeling methods. Antibodies that perform well on tissue sections produced similar quality results in various organs and thick specimens. Labeling relatively thick sections (30–100 μm) with antibodies using existing methods takes 1–2 days or longer. PRESTO markedly shortened the incubation time to 2–3 hours. In addition, the PRESTO technique is applicable to intact thick tissues without eliminating lipid. Applying c-PRESTO or s-PRESTO substantially expedited antibody penetration into thick brain slices without ACT, and results after 2 hours of PRESTO were superior to 2 days of passive incubation. In contrast, PRESTO was of no benefit for ACT-processed brain slices, suggesting that PRESTO helps macromolecules penetrate into dense tissues. The PRESTO procedure can be easily implemented with other tissue clearing methods, such as SeeDB, which require antibody labeling before tissue clearing. For example, brain tissues can be pre-labeled with PRESTO and visualized by SeeDB (Supplementary Fig. 8b).

In conclusion, the ACT-PRESTO method provides a reliable quick way to clear thick specimens and label deep structures in intact organs or even a whole body. This method is compatible with standard immunolabeling procedures, as we demonstrated with a panel of 82 antibodies. This method significantly improves optical transparency of tissues and facilitates immunolabeling and tissue clearing techniques will be easily accessible to most laboratories. When combined with the latest imaging technology, such as light-sheet and super-resolution microscopy, ACT-PRESTO should advance molecular and cellular analyses of normal and diseased organs.

## Materials and Methods

### Specimen Preparation

All animal husbandry and all animal care and euthanasia as described were in accordance with guidelines from the Korea University and have been approved by members of the Korea University Institutional Animal Care and Use Committee.

*Mice,* Male C57BL/6 mice were purchased from DH Biolink, Inc. (Seoul, Korea). Mice that were 21 days- and 2-months-old were used for whole-body and organ clearing, respectively. Mice were transcardially perfused with 40 ml 0.1 M PBS (pH 7.4) followed by 50 ml 4% PFA in 0.1 M PBS. Organs from adult mice were post-fixed in 4% PFA overnight at 4 °C. Fixed samples were incubated in A4P0 hydrogel monomer solution (4% acrylamide in 0.1 M PBS) supplemented with 0.25% of the photoinitiator 2,2′-azobis[2-(2-imidazolin-2-yl)propane] dihydrochloride (Wako Pure Chemical, Osaka, Japan) overnight at 4 °C. Hydrogel-infused samples were de-gassed for 5 min and polymerized for 2–3 hours at 37 °C. Some samples were washed in 0.01 M PBS before ACT clearing (see Supplementary Table 4).

*Rats,* Adult male Sprague–Dawley rats (12–16-weeks-of-age) were anesthetized deeply with urethane (100 mg/kg) and transcardially perfused with 0.1 PBS (pH 7.4), followed by 4% PFA in 0.1 M PBS. Brains from adult rats were post-fixed in 4% PFA for 1 day at 4 °C. Fixed brains were incubated in A4P0 hydrogel monomer solution supplemented with 0.25% of the photoinitiator 2,2′-azobis[2-(2-imidazolin-2-yl)propane] dihydrochloride for 2 days at 4 °C. The hydrogel-infused rat brains were de-gassed for 5 min and polymerized for 2–3 hours at 37 °C. After polymerization, the brains were washed in 0.01 M PBS before ACT clearing.

*Rabbits,* Specific pathogen-free adult male white New Zealand rabbits (weight, 3.0–3.5 kg; (Orient Animal Co. Ltd., Jiangsu, China) were fixed by transcardial perfusion. The brains were post-fixed in 4% PFA for 2 days and then incubated in A4P0 for 3 days at 4 °C.

*Zebrafish,* Zebrafish (age, 160–180 days) were kindly gifted by Professor Hae Chul Park (Korea University). The fish were euthanized in tricaine (ethyl 3-aminobenzoate methane sulfonate salt; Sigma-Aldrich, St. Louis, MO, USA), fixed in 4% PFA for 2–3 hours at 4 °C and incubated in A4P0 overnight at 4 °C. ACT-processed zebrafish were transferred to a clean dish or glass beaker, incubated for 30 min in bleach solution (0.15 mg potassium permanganate and 0.3% sulfuric acid in 50 ml dH_2_O), washed with 0.01 M PBS, and incubated in 1% oxalic acid solution until colorless.

*Xenopus, Xenopus tropicalis* frogs (age, 3 months) were kindly gifted by Professor Hosung Jung (Yonsei University). The frogs were euthanized in tricaine, fixed overnight in 4% PFA, washed with 0.01 M PBS, and then incubated in A4P0 for 1 day at 4 °C.

*Chicken,* Fertilized chicken eggs (Phulmuone Foods, Seoul, Korea) were incubated at 38 °C in a humidified incubator until the appropriate stage^37^ of 7–12-days-old (Supplementary Fig. 6). After fixation in 4% PFA, the embryos were incubated in A4P0 solution overnight at 4 °C.

*Octopus,* Small octopi were obtained from a local fishery and immersion-fixed in 4% PFA for 2 days, washed with 0.01 M PBS overnight, and immersed in A4P0 for 1 day at 4 °C.

*Human autopsy tissues,* a cadaver for medical student education with no history of spinal surgery or deformity was obtained under Korea University Anatomical Donation Program (KUADP) and treated in accordance with an accurate observance of the university guidelines. We got an informed consent from the donator and the procedures were approved by Cadaver research Institutional Review Board of Korea University College of Medicine. The cadaver was perfused with 10% formalin in saline and the upper cervical part of spinal cord was dissected out, divided into several blocks, and incubated in A4P0 for 3 days at 4 °C.

### ACT Reagents

*A4P0 solution,* Forty ml of 40% acrylamide was added to 360 ml dH_2_O to prepare 400 ml of the hydrogel monomer solution (A4P0). The VA-044 initiator (100 mg) was added to 40 ml of hydrogel monomer solution (0.25%) in a 50 ml conical tube under a fume hood immediately prior to use.

*ETC buffer,* Sodium dodecyl sulfate (SDS; 40 g) and 200 mM boric acid were added to dH_2_O to prepare 1 L of ETC buffer, and pH was adjusted to 8.5.

*RIMS,* Histodenz (40 g) was dissolved in 0.02 M phosphate buffer (pH 7.5) and was brought up to 30 ml with 0.01% sodium azide.

*CUBIC-mount,* Sucrose (250 g of 50%, w/v), 125 g urea (25%, w/v), and 125 g N,N,N′,N′-tetrakis(2-hydroxypropyl)ethylenediamine (25%, w/v) were dissolved in 150 ml of dH_2_O and brought up to 500 ml.

Detailed information on the reagents is provided as Supplementary Table 1.

### ACT Clearing

Details of the ACT-PRESTO protocol are provided in Supplementary Information. Briefly, polymerized samples (see Specimen Preparation section) were transferred to the tissue container in the ETC chamber (Supplementary Table 3). The tissue container holder was placed in the chamber containing 4% SDS in 200 mM boric acid in H_2_O (pH 8.5), and the ETC conditions (current/temperature/current direction switching time) were run. The ETC conditions were adjusted depending on specimen size (Supplementary Table 4). Cleared samples were washed in 0.01 M PBS overnight at room temperature with gentle shaking to remove the remaining SDS.

### Three-dimensional Immunostaining of ACT Brain Samples

Cleared 1-mm brain slices were washed several times with 0.01 M PBS at room temperature with gentle shaking, and then incubated with primary antibodies (see Supplementary Table 3) in 3% (w/v) bovine serum albumin (BSA), 0.1% (v/v) Triton X-100, and 0.01% (w/v) sodium azide in 0.1 M PBS overnight at 37 °C with shaking. The stained samples were washed several times with 0.01 M PBS at room temperature with gentle shaking and then stained with secondary antibodies in 3% (w/v) BSA, 0.1% (v/v) Triton X-100, and 0.01% (w/v) sodium azide in 0.1 M PBS overnight 37 °C with gentle shaking. The stained samples were washed several times with 0.01 M PBS under gentle shaking and immersed in RIMS or CUBIC-mount solution overnight at room temperature (Supplementary Figs 3 and 4).

Whole-mouse brain and spinal cord were immunostained with primary antibodies in 6% (w/v) BSA, 0.1% (v/v) Triton X-100, and 0.01% (w/v) sodium azide in 0.1 M PBS for 2 days at 37 °C with shaking. The stained samples were washed several times with 0.01 M PBS at room temperature with gentle shaking and then stained with secondary antibodies in 6% (w/v) BSA, 0.1% (v/v) Triton X-100, and 0.01% (w/v) sodium azide in 0.1 M PBS for 2 days 37 °C with gentle shaking. The stained samples were washed several times with 0.01 M PBS under gentle shaking and immersed in RIMS or CUBIC-mount solution for 1 day at room temperature (Fig. 2a, Supplementary Fig. 2b).

### Whole-body and Organ ACT Protocol

We slightly modified the ACT protocol described above to improve tissue quality for whole-body and organ imaging. Adult (8–9-week-old) mice were fixed by transcardial perfusion, and then immersed in A4P0 solution for 2 days. The clearing solution was prepared in 0.01 M PBS, rather than H_2_O, to maintain the shape of the body and decrease clearing time. Higher sodium concentrations in the clearing solution (0.03 M and 0.1 M PBS) ameliorated tissue swelling but resulted in tissue thinning (data not shown). Therefore, we chose 0.01 M PBS for the whole body, organs, and embryos. After ETC, the samples were washed several times with 0.01 M PBS before immunostaining. A 6-week-old mouse was fixed by transcardial perfusion, and the head was excised for whole head imaging (Fig. 6b). The head was freed from subcutaneous skin and hair and washed several times with 0.1 M PBS. The cleaned head was post-fixed and cleared in 0.01 M PBS-based clearing solution. After clearing, the whole body, head, and organs were stained with antibodies using the s-PRESTO method (see below).

### Deep immunolabeling with PRESTO (organs and head)

*c-PRESTO*, A cleared sample was transferred to an e-tube. After adding 400 μl of the primary antibody diluted solution (1:100–500, in 0.1 M PBS containing 6% BSA and 0.1% Triton X-100), the e-tube was centrifuged at 600 × g for 3 hours. The stained sample was washed with 0.01 M PBS by centrifugation at 600 × g for 30 min and then stained with fluorescently conjugated secondary antibody (1:100–500 dilution in 0.1 M PBS containing 6% BSA and 0.1% Triton X-100) for 3 hours (Fig. 5c,d).

*s-PRESTO,* A sample was transferred to a syringe filled with 2–5 ml of primary antibody solution (1:500–800, in 0.1 M PBS containing 6% BSA and 0.1% Triton X-100). We set the infusion/withdrawal volume and speed at 10 ml/min and a 4 min pause time on continuous cycle mode (see ACT-PRESTO protocol). The pumps were infused and the direction of flow was changed when the target volume was reached (withdrawal) after a brief pause. The syringe pump ran for 3–24 hours at room temperature (Fig. 6). Immunolabeled tissue was washed four times with 0.01 M PBS and stained with fluorescently conjugated secondary antibody (1:100–500 dilution in 0.1 M PBS containing 6% BSA and 0.1% Triton X-100) for 3–24 hours using the syringe pump.

### Fluorescence microscopy

Cleared tissue samples were incubated in RIMS or CUBIC-mount solution for several hours to days depending on tissue size. The samples were mounted in RIMS or CUBIC-mount solution using 35-mm glass-bottom dished (100350; SPL Lifesciences Inc., Seoul, South Korea). Coverslipped samples were stored at room temperature and protected from light prior to imaging. Most fluorescence images were visualized initially under a conventional confocal microscope (Carl Zeiss LSM 700; Carl Zeiss Inc., Zena, Germany) with either the Fluar 5 × (NA = 0.25, working distance = 12.5 mm)or Plan-Apochromat 10 × (NA = 0.45, working distance = 2.1 mm) objective lenses. Spinal cord images were taken on a two-photon microscope (Carl Zeiss LSM 710 NLO) with the Plan-Apochromat 10 × (NA = 0.45, working distance = 2.1 mm) objective lens (Supplementary Fig. 2b, *top*). Laser output was increased gradually according to Z-stack acquisition to detect most of the stained signals from the mouse brain slices and whole-head and whole-organ fluorescence images in the unsaturated intensity range. Mouse whole-brain and spinal cord fluorescence images were acquired with a light-sheet fluorescence microscope (light-sheet Z1; Carl Zeiss) combined with 405 nm and 568 nm lasers and the Clr Plan-Neofluar 20 × /1.0 lens (corrected nd = 1.45). Samples were immersed in RIMS during image acquisition. Images were captured using 1 × zoom (for whole brain) and 2× zoom (for spinal cord) under a light-sheet microscope (Fig. 2a and Supplementary Fig. 2b). We used a customized sample holder to capture mouse whole-brain and spinal cord images. Each plane was illuminated from the left and right sides, and the merged image was saved. After imaging, the samples were stored in RIMS at room temperature and protected from light.

### Image Processing

We used ZEN software (Carl Zeiss) for 3D reconstruction of most images. We obtained 3D reconstructions from Z-stacks consisting of 100–700 optical slices taken at intervals of 1–5 μm. We applied 3D view volume imaging on the image stacks used for the high-resolution images with ZEN software (2011 Black edition). We also used Vaa3D image software for volume image testis (www.vaa3d.org). The 2D image series obtained from the Zeiss LSM700 microscope were stacked automatically using the callback function and rendered to 3D volume images in 3D view plugin. A snapshot of the 3D image was captured. The 3D-rendered single neuron images were visualized and captured with Avizo ver. 5.0 software (FEI, Hillsboro, OR, USA). Whole-mouse brain and spinal cord images generated with the Z.1 light-sheet microscope (Zeiss) were aligned into large comprehensive image stacks and converted into movies using Arivis Vision 4D software (Arivis AG, Rostock, Germany).

### Viral Injection

Adult male mice (weight, 20–22 g) were deeply anesthetized with 50 mg/kg sodium pentobarbital and placed in a stereotaxic device (Stoelting, Wood Dale, IL, USA) for stereotaxic injection of AAV-GFP (AAV-GFP serotype 6; UNC Vector Core, Chapel Hill, NC, USA), and the AAV-GFP-virus was injected into the striatum (AP: + 0.2 mm, ML: −1.4 mm, DV: −2.2 mm to Bregma) using a 30-gauge micro-syringe at a rate of 0.2 μl/min. The needle was slowly withdrawn 15 min later. Virus-injected mice were killed 3 days after injection.

### Tissue Transparency and Size Change Measurements

Fixed adult mouse brains were cut into 1-mm-thick coronal slices. The slices were weighed and imaged with a conventional camera before and after clearing. We measured the size and transparency of the cleared 1 mm-thick brain slices, which were outlined and calculated using Adobe Photoshop software (Adobe Systems Inc., San Jose, CA, USA). We used the gray value of the cleared sample image to measure tissue transparency, and transparency was normalized to the uncleared counterpart (Supplementary Fig. 2a,c). Increased tissue size was determined by calculating the size change of the slice before and after clearing and presented as a percentage of the pre-cleared size. Recovery of over-clearing tissue size was measured by calculating the size change of the slice immediately after ACT processing and after matching the RI in RIMS (Supplementary Figs 2d and 3c).

### Antibody Diffusion Rate Measurement

Adult mouse brain (ACT processed brain and uncleared) and various organ (4 × 4 × 15 mm) samples were rinsed in 0.01 M PBS. The samples were incubated with a secondary antibody conjugated to a fluorescent label (1:300, Alexa Cy3; 568 nm) in 6% BSA/0.1% Triton X-100 in 0.1 M PBS. The samples were cut into 2-mm thick slices, and imaged in 0.1 M PBS using an EVOS microscope (Life Technologies, Carlsbad, CA, USA). An intensity plot was generated using ImageJ to measure the degree of antibody penetration (Fig. 5a).

### Clearing In-gel Tissue

Micro-punches (2-mm diameter) of intact and polymerized tissue-hydrogels were obtained from a 1-mm brain slice and placed in a Western gel caster (0.7-mm spacer), and 6% acrylamide solution (24:1 acrylamide: bis-acrylamide in ACT running buffer) was poured into the cast and polymerized. The polymerized gel was electrophoresed with the mini-PAGE gel electrophoresis system (Hoefer Inc. Holliston, MA, USA) at 100 V for 30 min using ACT running buffer. The gel containing the tissue-hydrogels was immersed in Coomassie Blue staining solution for 30 min and de-stained in acetic acid solution overnight.

### Protein Loss Measurement

Three adult mouse (age, 8-weeks) brains (control) and two hydrogel-embedded brains from each condition (CLARITY and ACT) were cut into 1-mm coronal blocks. The brain blocks were placed in the ETC chamber containing clearing solution. The tissues were cleared for 2 hours, and the quantity of protein lost from the tissue into the clearing solution was measured using the bicinchoninic acid protein assay. Total mouse brain protein content was estimated to be 10% (wt) based on a previous report^38^.

### *In situ* Hybridization

One-mm brain slices were fixed for 20 min with 4% PFA, and washed with 0.1 M PBS for *in situ* hybridization^39^. The acetylation reaction (0.25% acetic anhydride in 0.1 M RNase-Free triethanolamine-HCl, pH 8.0) was performed for 10 min at room temperature. After washing with 0.1 M PBS, the brain slices were dipped in pre-hybridization buffer for 2 hours at 60 °C and hybridized with 1–2 μg/ml DIG-labeled riboprobe (Mm-TH # NM_009377.1; F: 5′-GCTTCTCTGACCAGGCGTAT-3′, R: 5′-CACTCAGTGCTTGGGTCAGG-3′) overnight at 60 °C. The brain slices were washed consecutively in wash buffer (50% formamide, 0.2 × SSC, and PBST (0.1% Triton X-100 in 0.1 M PBS) at 60 °C. After blocking with 10% sheep serum in PBST for 1 hour, anti-DIG-AP (1:2000; Roche, Manheim, Germany) was applied to the brain slices overnight at room temperature. After two washes with PBST, the samples were washed quickly with 0.1 M PBS. The brain slices were incubated in NBP/BCIP developing solution for 8 hours overnight to develop the signal. The brain sections were dipped in tap water to stop the reaction. After three washes in buffer, the brain slices were placed in RIMS, and observed using the EVOS microscope (Fig. 2c).

## Additional Information

**How to cite this article**: Lee, E. *et al.* ACT-PRESTO: Rapid and consistent tissue clearing and labeling method for 3-dimensional (3D) imaging. *Sci. Rep.* **6**, 18631; doi: 10.1038/srep18631 (2016).

## Supplementary Material

Supplementary Information

Supplementary Video 1

Supplementary Video 2

Supplementary Video 3
