# Corrigendum: ACT-PRESTO: Rapid and consistent tissue clearing and labeling method for 3-dimensional (3D) imaging

**DOI:** 10.1038/srep31940

**Published:** 2016-09-01

**Authors:** Eunsoo Lee, Jungyoon Choi, Youhwa Jo, Joo Yeon Kim, Yu Jin Jang, Hye Myeong Lee, So Yeun Kim, Ho-Jae Lee, Keunchang Cho, Neoncheol Jung, Eun Mi Hur, Sung Jin Jeong, Cheil Moon, Youngshik Choe, Im Joo Rhyu, Hyun Kim, Woong Sun

Scientific Reports 6: Article number: 18631; 10.1038/srep18631 published online: 01 11 2016; updated: 09 01 2016.

In this Article and Supplementary Information file, some instances of ‘0.001M PBS’ and ‘0.01M PBS’ are incorrectly written as ‘0.01M PBS’ and ‘0.1M PBS’ respectively. With the exception of the following:

In the Materials and Methods section, under subheading ‘Specimen Preparation’,

“Fixed samples were incubated in A4P0 hydrogel monomer solution (4% acrylamide in 0.1 M PBS) supplemented with 0.25% of the photoinitiator 2,2′-azobis[2-(2-imidazolin-2-yl)propane] dihydrochloride (Wako Pure Chemical, Osaka, Japan) overnight at 4 °C”.

should read:

“Fixed samples were incubated in A4P0 hydrogel monomer solution (4% acrylamide in 0.001 M PBS) supplemented with 0.25% of the photoinitiator 2,2′-azobis[2-(2-imidazolin-2-yl)propane] dihydrochloride (Wako Pure Chemical, Osaka, Japan) overnight at 4 °C”.

Under subheading ‘ACT Reagents’,

“*A4P0 solution,* Forty ml of 40% acrylamide was added to 360 ml dH_2_O to prepare 400 ml of the hydrogel monomer solution (A4P0)”.

should read:

“*A4P0 solution,* Forty ml of 40% acrylamide was added to 360 ml 0.001M PBS to prepare 400 ml of the hydrogel monomer solution (A4P0)”.

Under subheading ‘Whole-body and Organ ACT Protocol,

“Higher sodium concentrations in the clearing solution (0.03 M and 0.1 M PBS) ameliorated tissue swelling but resulted in tissue thinning (data not shown)”.

should read:

“Higher sodium concentrations in the clearing solution (0.003 M and 0.01 M PBS) ameliorated tissue swelling but resulted in tissue thinning (data not shown)”.

“The head was freed from subcutaneous skin and hair and washed several times with 0.1 M PBS.

should read:

“The head was freed from subcutaneous skin and hair and washed several times with 0.001 M PBS”.

In the Supplementary Information file, under subheading ‘’Hydrogel monomer infusion & polymerization,

“1. Prepare the A4P0 hydrogel monomer solution.

Take 40 ml of 4% acrylamide in 0.1 M PBS solution and add 100 mg of VA-044 Initiator to make a final concentration of 0.25%”.

should read:

“1. Prepare the A4P0 hydrogel monomer solution.

Take 40 ml of 4% acrylamide in 0.001 M PBS solution and add 100 mg of VA-044 Initiator to make a final concentration of 0.25%”.

Under the subheading ‘Immunolabeling ACT-processed brain tissue’,

“1. Wash samples in 0.1 M PBS for 3–5 hours and change the buffer every hour”.

should read:

“1. Wash samples in 0.001 M PBS for 3–5 hours and change the buffer every hour”.

“3. Wash samples in 0.1 M PBS for 3–5 hours, and change buffer every hour”.

should read:

“3. Wash samples in 0.001 M PBS for 3–5 hours, and change buffer every hour”.

