# Supplementary material for: ACT-PRESTO: Rapid and consistent tissue clearing and labeling method for 3-dimensional (3D) imaging
Source: Sci Rep. 2016 Jan 11;6:18631. doi: 10.1038/srep18631 (PMC4707495; doi:10.1038/srep18631)
Supplement: Supplementary Information [file srep18631-s1.pdf]

ACT-PRESTO: Rapid and consistent tissue clearing and labeling method for 3-dimensional (3D) imaging

Eunsoo Lee[1], Jungyoon Choi[1], Youhwa Jo[1], Joo Yeon Kim[1], Yu Jin Jang[2], Hye Myeong Lee[2], So Yeun Kim[3], Ho-Jae Lee[4], Keunchang Cho[4], Neoncheol Jung[4], Eun Mi Hur[5,6], Sung Jin Jeong[2], Cheil Moon[3], Youngshik Choe[2], Im Joo Rhyu[1], Hyun Kim[1], Woong Sun[1]

[1]Department of Anatomy and Division of Brain Korea 21 Plus Program for Biomedical Science, Korea University College of Medicine, Anam-dong, Seongbuk-gu, Seoul 136-705, Korea; [2]Department of Neural Development and Disease, Korea Brain Research Institute, 701-300 Daegu, Korea; [3]Department of Brain & Cognitive Sciences, Graduate School, Daegu Gyeungbuk Institute of Science and Technology (DGIST), Daegu, Korea; [4]Logos Biosystems, Inc. Anyang-Si, Gyunggi-Do, 431-755, Republic of Korea; [5]Center for Neuroscience, Brain Science Institute, Korea Institute of Science and Technology, Seoul, Korea, [6]Department of Neuroscience, Korea University of Science and Technology (UST), Daejeon, Korea.

Corresponding author:

Woong Sun, Ph.D

Department of Anatomy, Korea University College of Medicine,

Anam-dong, Seongbuk-gu, Seoul 136-705, Korea

E-mail: woongsun@korea.ac.kr

Tel: +82-2-2286-1404, Fax: +82-2-929-5696

Supplementary information

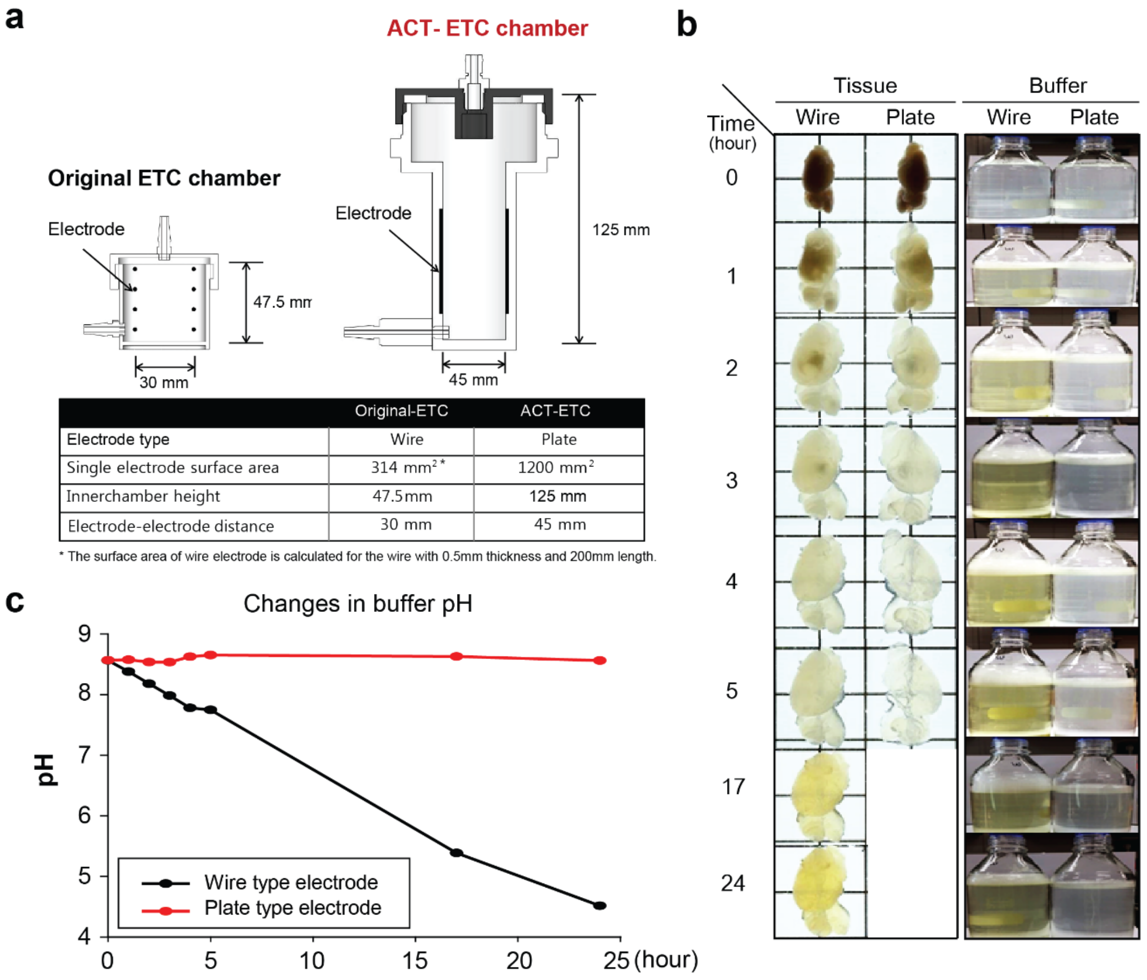

	Original-ETC	ACT-ETC
Electrode type	Wire	Plate
Single electrode surface area	314 mm^{2}*	1200 mm^2
Innerchamber height	47.5mm	125 mm
Electrode-electrode distance	30 mm	45 mm

* The surface area of wire electrode is calculated for the wire with 0.5mm thickness and 200mm length.

Supplementary Figure 1. Comparison of electrophoretic tissue clearing (ETC) systems with the CLARITY technique.

(a) Dimensions of the Active Clarity Technique (ACT)-ETC chamber containing the electrode area, electrode-electrode distance, and inner-chamber dimensions. **(b)** Comparison between tissue and buffers used for clearing and the original and ACT-ETC chambers. **(c)** Changes in pH of the clearing buffers during ETC.

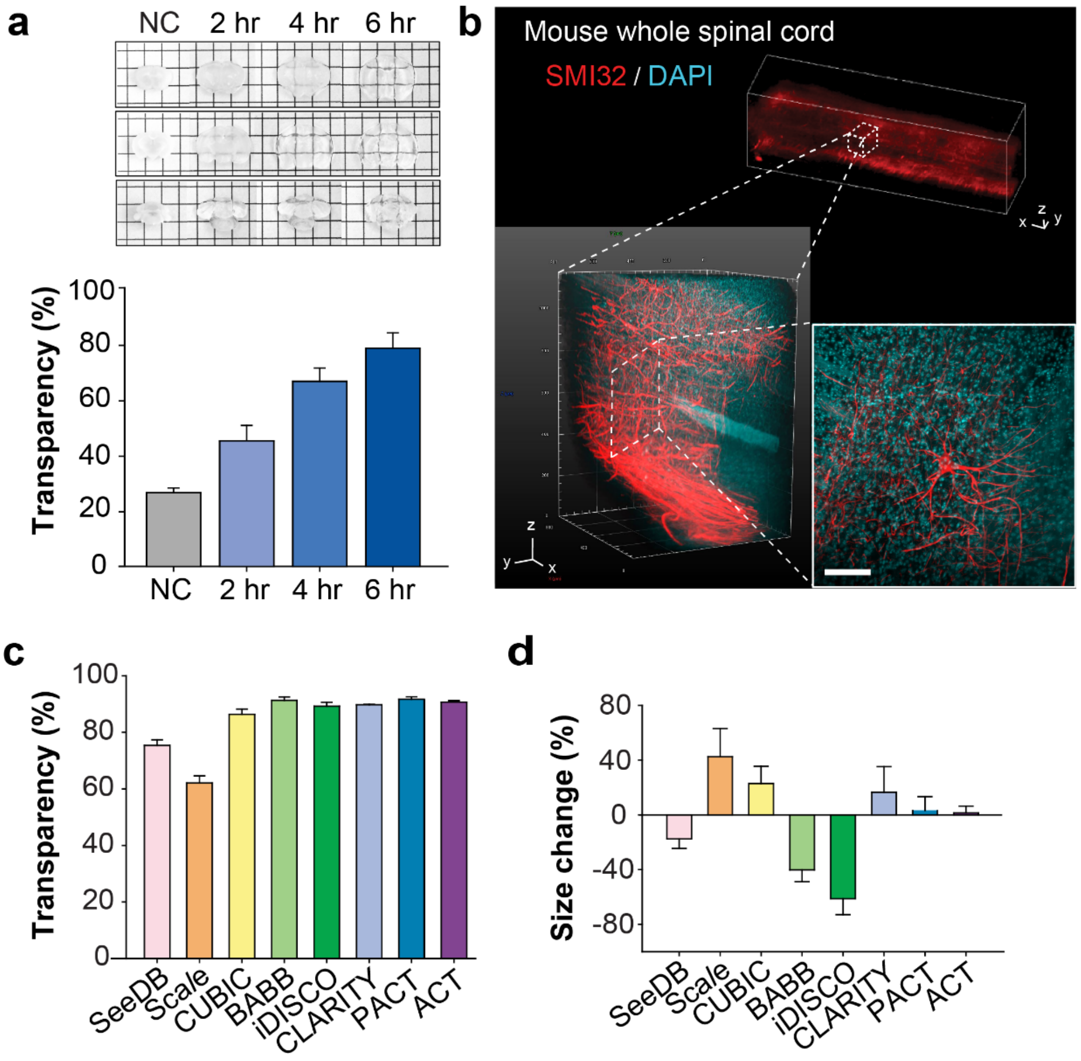

Supplementary Figure 2. Efficient clearance of Active Clarity Technique (ACT)-processed tissue

(a) Clearing efficiency of adult whole mouse brains over tissue clearing time by the ACT. After clearing, whole brains were cut into 2-mm thick coronal slices to measure transparency. Square unit, x: 5 mm, y: 5 mm. **(b)** Two-photon imaging of a whole spinal cord labeled with SMI32 antibody and nuclei counter-stained with DAPI (10× objective; stack size, 860 µm; step size,

2 µm). Inset shows a high magnification image taken by the Z.1 light-sheet microscope (20× objective; stack size, 1,188 µm; step size, 2 µm). Scale bar, 100 µm. **(c, d)** Comparison of ACT with other clearing methods. **(c)** Transparency of cleared slices is normalized to the blank, defined as 100. **(d)** Size changes after clearing. Images were taken in the refractive index matching solution (RIMS).

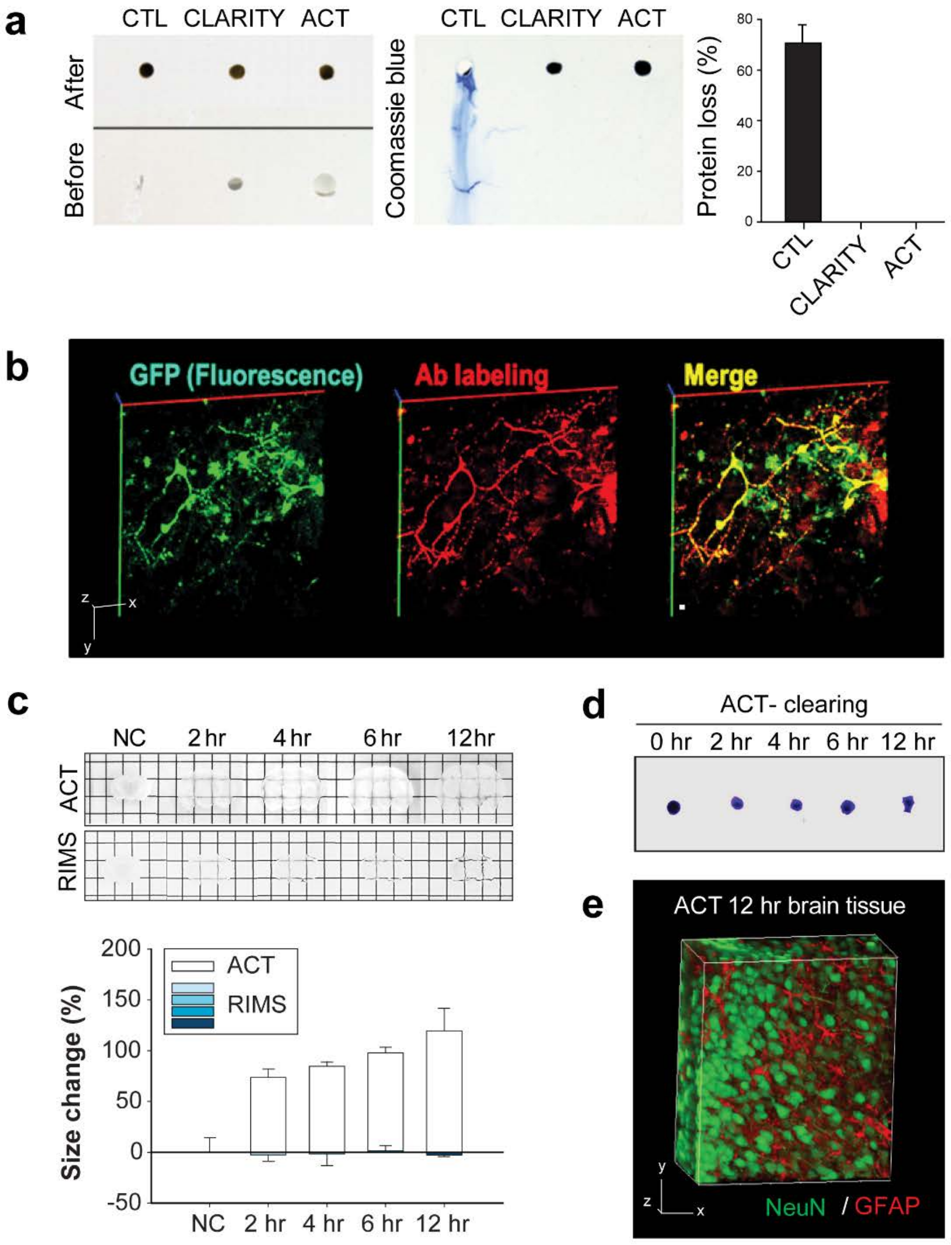

Supplementary Figure 3. Active Clarity Technique (ACT) maintains fluorescence protein signals and detailed structure.

(a) In-gel electrophoresis of control (CTL), CLARITY-, and ACT-processed hydrogels. Micro-

punched tissue-hydrogels were further in-gel electrophoresed for 1 hour and the proteins were extracted. After electrophoresis, the tissue-embedded gel was stained with Coomassie Blue. The measurement of protein loss by conventional methods is shown as a graph (n = 3 slices/clearing condition). Data = Mean ± SD. **(b)** Presence of viral green fluorescent protein (GFP) fluorescence was determined by the GFP antibody-labeled signal (red). One-mm cleared brain slice (10× objective; stack size, 200 μm, step size, 1 μm). Scale bar, 100 μm. **(c)** 1mm brain slices were cleared for 2–12 hours and the size change is presented for expansion in ACT and their recovery in refractive index matching solution (RIMS) (n = 8-fields of view/sample). **(d)** Protein loss in over-cleared tissue (0–12 hours of ACT) was not detected, as shown by Coomassie Blue staining of the punched tissue-hydrogel without loss of protein. **(e)** Immunoreactivity was maintained in the over-cleared tissue. The ACT-processed tissue (12 hours) was stained for glial fibrillary acidic protein (GFAP, red)/neuronal marker (NeuN, green) (10× objective, 2× confocal zoom; stack size, 220 μm, step size, 2 μm). Scale bar, 50 μm.

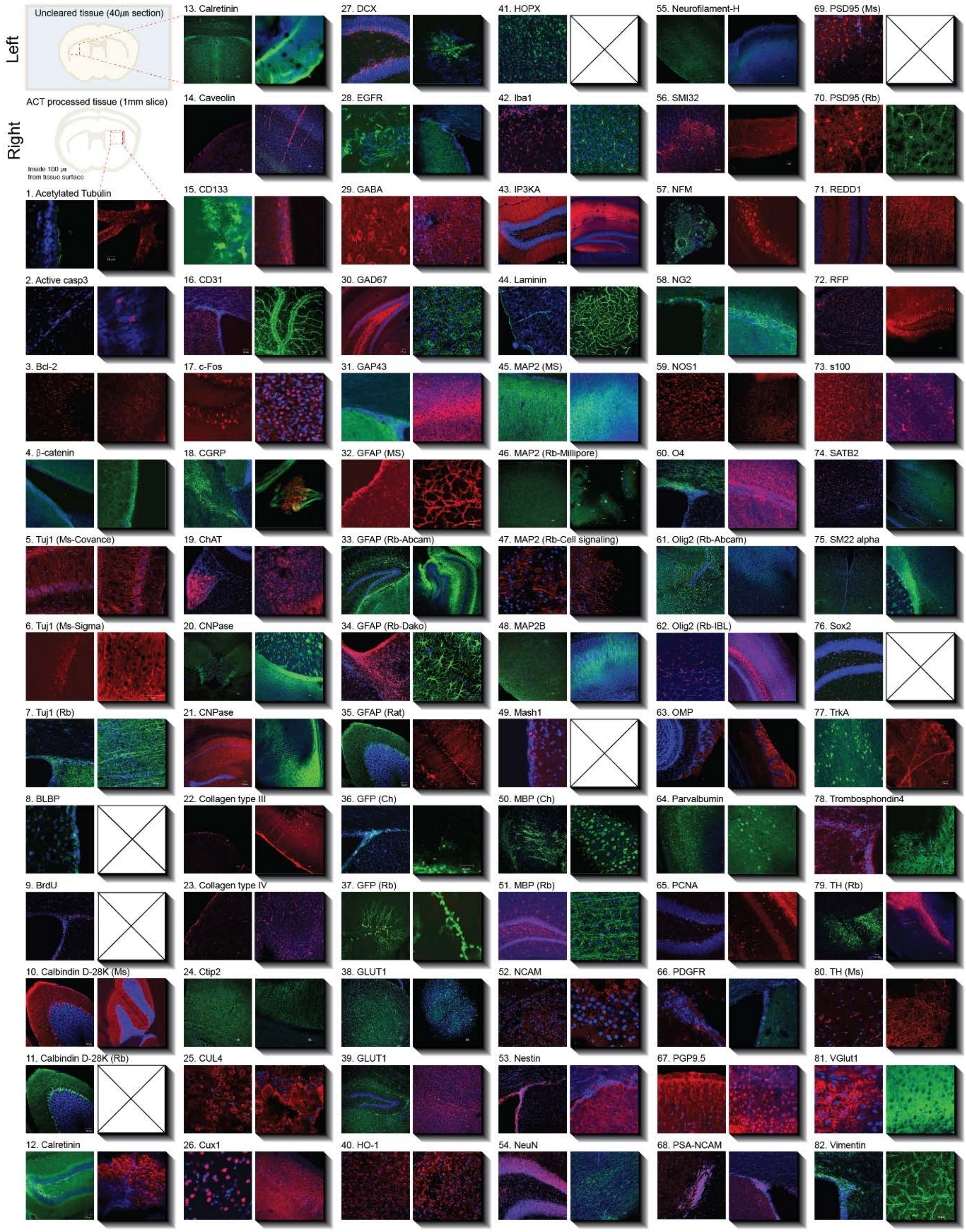
Left
Uncleared tissue (40μm section)
Right
ACT processed tissue (1mm slice)
Inside 100 μm from tissue surface
1. Acetylated Tubulin
2. Active casp3
3. Bcl-2
4. β-catenin
5. Tuj1 (Ms-Covance)
6. Tuj1 (Ms-Sigma)
7. Tuj1 (Rb)
8. BLBP
9. BrdU
10. Calbindin D-28K (Ms)
11. Calbindin D-28K (Rb)
12. Calretinin
13. Calretinin
14. Caveolin
15. CD133
16. CD31
17. c-Fos
18. CGRP
19. ChAT
20. CNPase
21. CNPase
22. Collagen type III
23. Collagen type IV
24. Ctip2
25. CUL4
26. Cux1
27. DCX
28. EGFR
29. GABA
30. GAD67
31. GAP43
32. GFAP (MS)
33. GFAP (Rb-Abcam)
34. GFAP (Rb-Dako)
35. GFAP (Rat)
36. GFP (Ch)
37. GFP (Rb)
38. GLUT1
39. GLUT1
40. HO-1
41. HOPX
42. Iba1
43. IP3KA
44. Laminin
45. MAP2 (MS)
46. MAP2 (Rb-Millipore)
47. MAP2 (Rb-Cell signaling)
48. MAP2B
49. Mash1
50. MBP (Ch)
51. MBP (Rb)
52. NCAM
53. Nestin
54. NeuN
55. Neurofilament-H
56. SMI32
57. NFM
58. NG2
59. NOS1
60. O4
61. Olig2 (Rb-Abcam)
62. Olig2 (Rb-IBL)
63. OMP
64. Parvalbumin
65. PCNA
66. PDGFR
67. PGP9.5
68. PSA-NCAM
69. PSD95 (Ms)
70. PSD95 (Rb)
71. REDD1
72. RFP
73. s100
74. SATB2
75. SM22 alpha
76. Sox2
77. TrkA
78. Trombosphondin4
79. TH (Rb)
80. TH (Ms)
81. VGlut1
82. Vimentin

Supplementary Figure 4. Versatility of Active Clarity Technique (ACT)-processed tissues for antibody staining

Images of commercially available antibodies (Table S2) applied to ACT-cleared tissues. *Left*. Images show antibody staining of uncleared thin sections (40 μm). *Right*. Images of cleared (1 mm) brain slices by ACT. The ACT-processed tissue images were taken at a depth of ~100 μm from the surface of the brain slice. A total of 75 of 82 antibodies worked in ACT-processed tissues. All images were processed with an identical contrast threshold. Objectives used for confocal imaging: Zeiss LSM 700, objective; Plan-Apochromat 10× (NA = 0.45, working distance = 2.1 mm).

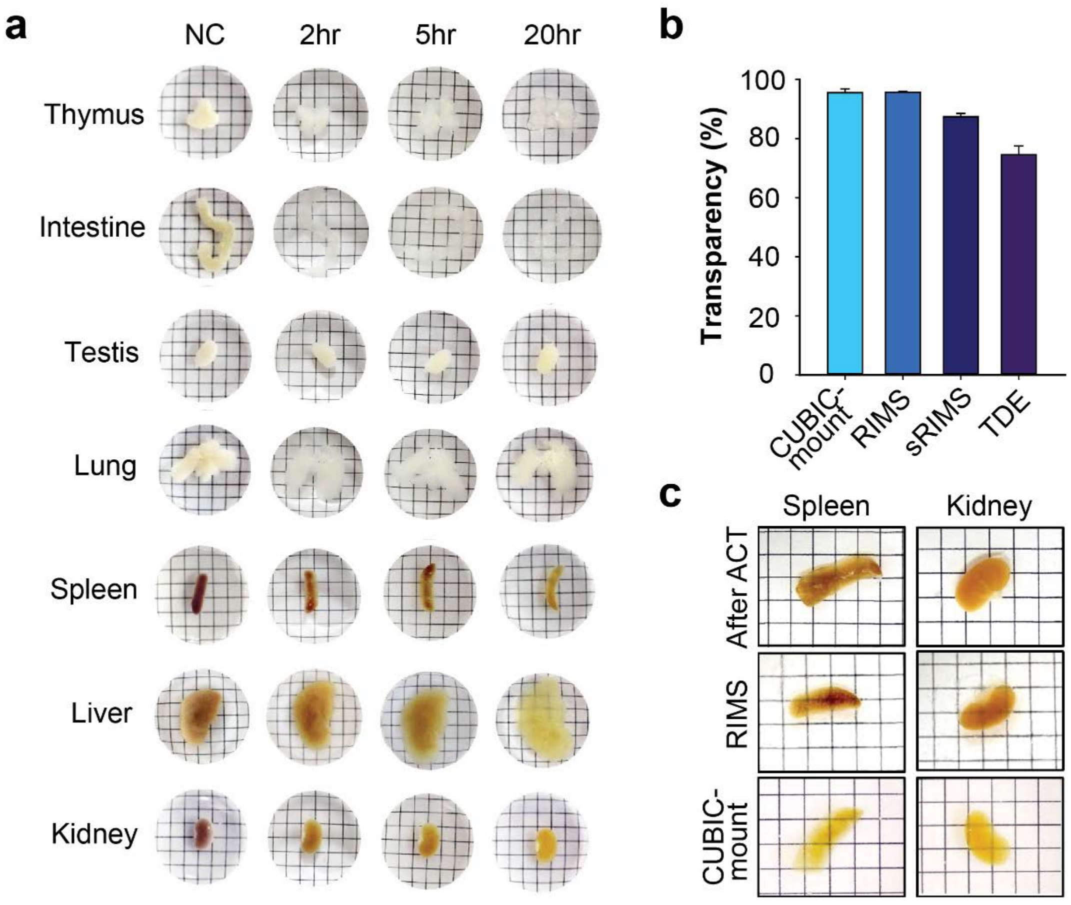

Supplementary Figure 5. Applying of the Active Clarity Technique (ACT) for whole organ clearing and decolorizing

(a) Representative images of whole mouse organs (thymus, intestine, testis, lung, spleen, liver, and kidney) during ACT clearing. Spleen, liver, and kidney maintained an opaque color after clearing. Digital images of the samples were taken using a bright-field camera (square unit; x: 5 mm, y: 5 mm). **(b)** Comparison of optical transparency in CUBIC-mount with other refractive index-matching solutions. **(c)** Comparison of optical transparency of whole-mouse kidney and

spleen after a 2 day incubation in refractive index matching solution (RIMS) (middle panel) and CUBIC-mount (bottom panel). Square unit; x: 5 mm, y: 5 mm.

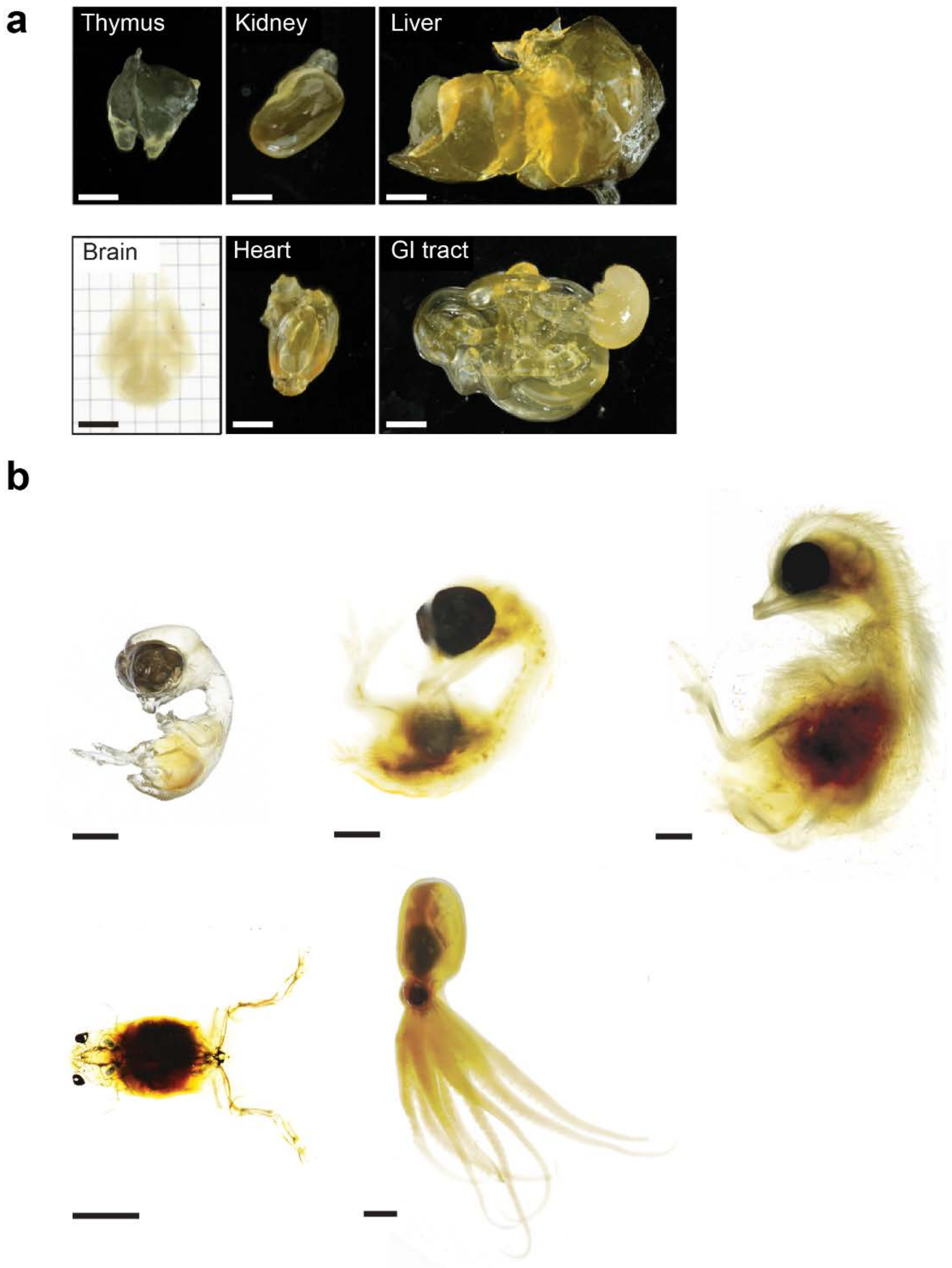

Supplementary Figure 6. Active Clarity Technique (ACT) is applicable to whole organisms

(a) Whole organs were collected from 2 day ACT-processed whole mouse bodies. Scale bar, 1 cm. **(b)** Vertebrate and invertebrate model animals, such as chickens, *Xenopus*, and the small

octopus were cleared by the ACT. Scale bar, 1 cm (see Supplementary Table 4 for ACT conditions and times).

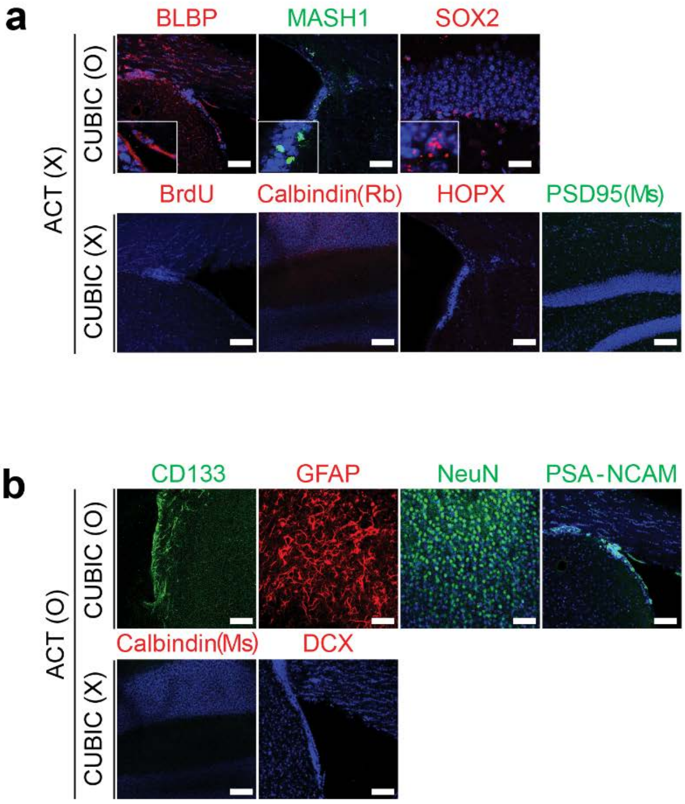

Supplementary Figure 7. Applicability of the CUBIC clearing method for antibodies

(a) Among the seven antibodies that did not work in Active Clarity Technique (ACT)-processed tissues (Supplementary Fig. 4), three (BLBP, Mash1, and Sox2) worked in CUBIC-processed tissues, Scale bar, 50 μm. **(b)** Antibodies chosen randomly from Supplementary Table 2 were tested on CUBIC-processed tissues. Calbindin (Ms) and DCX antibodies did not work in CUBIC-processed tissues. All images were processed with an identical contrast threshold. Images were taken at a depth of ~100 μm from the surface of the tissue using a Zeiss LSM 700 confocal microscope with the Plan-Apochromat 10× (NA = 0.45, working distance = 2.1 mm) objective lens. Scale bar, 50 μm.

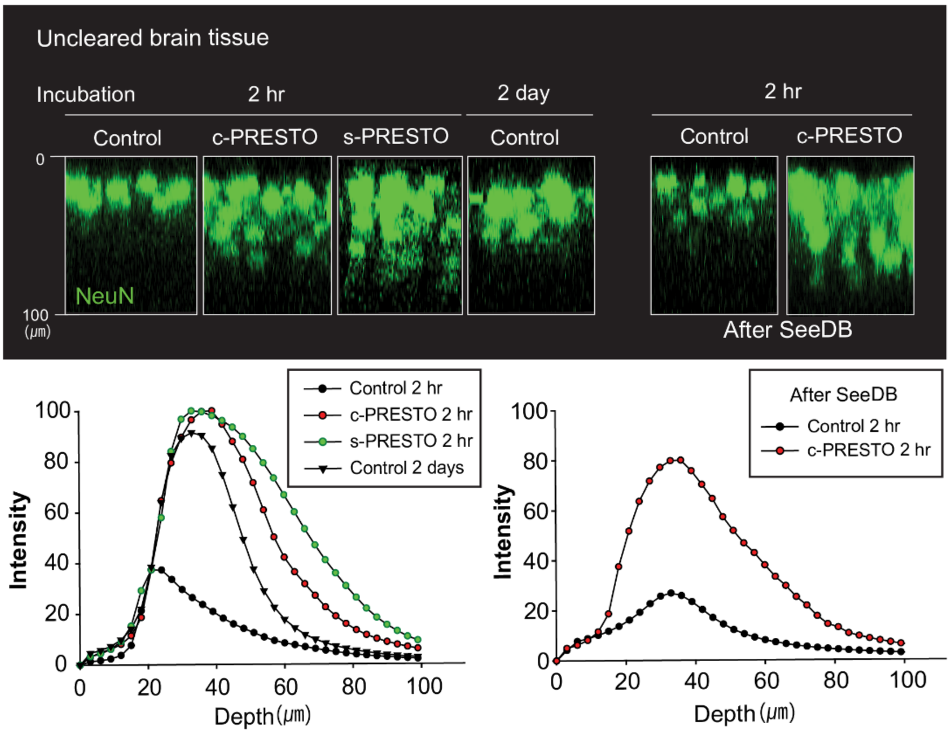

Supplementary Figure 8. Applicability of PRESTO for uncleared tissue

Uncleared mouse brain slices were stained for neuronal marker (NeuN) (green) using a PRESTO labeling technique. To compare antibody penetration, NeuN was stained in 1-mm adult mouse brain slices (uncleared sample) using a free diffusion antibody labeling method (control), c-PRESTO, or s-PRESTO for 2 hours. Fluorescence images before (*left* in black box) and after (*right* in black box) SeeDB. The tissues were centrifuged at 600 rcf for 2 hours using a table-top centrifuge during the primary (NeuN) and secondary antibody reaction for the c-PRESTO immunolabeling method. Syringe-(s)PRESTO uses a syringe pump (see online Methods) for the 2-hour NeuN antibody reaction. Uncleared brain tissues showed more than three-fold increased antibody penetration depth after 2 hours of s-PRESTO-, compared to the

control 2-hour processed tissue. The depth of the Z axis was > 90 μm in the s-PRESTO samples compared with 20–25 μm in control samples. Objectives used for confocal imaging: Zeiss LSM 700, objective; Plan-Apochromat 10× (NA = 0.45, working distance = 2.1 mm).

Supplementary Table 1. Reagent and material information related to the Active Clarity Technique (ACT)

Step		Material	company	Cat #
**Cardiac Perfusion-Fixation**		16% Paraformaldehyde	Lugen SCI	LGB-1175-16B
**Hydrogel monomer immersion**	Hydrogel monomer solution (A4P0)	40% (w/v) Acrylamide solution	Sigma-Aldrich	A4058
		Acrylamide	Affymetrix	75820
		2,2'-Azobis[2-(2-imidazolin-2-yl) propane] dihydrochloride	Wako Pure Chemical Industries	VA-044
**ACT**	Tissue clearing solution (4% SDS)	Boric acid	Affymetrix	76324
		Sodium Dodecyl Sulfate (SDS)	Affymetrix	18220
		Sodium hydroxide pellets	JUNSEI	1310-73-2
**Immuno-staining**	Antibody incubation buffer	Bovine Serum Albumin (BSA)	Santa Cruz Biotechnology	sc-2323A
		Triton X-100	Sigma-Aldrich	T8787
		Sodium azide	Sigma-Aldrich	S2002
	Secondary antibody	Alexa Fluor 488 Donkey anti-Chicken IgG (H+L)	Jackson ImmunoResearch	703-545-155
		Alexa Fluor 488 Donkey anti-Goat IgG (H+L)	Life Technologies - Molecular Probes	A11055
		Alexa Fluor 488 Donkey anti-Mouse IgG (H+L)	Life Technologies - Molecular Probes	A21202
		Alexa Fluor 488 Donkey anti-Rat IgG (H+L)	Life Technologies - Molecular Probes	A21208
		Alexa Fluor 488 Donkey anti-Rabbit IgG (H+L)	Life Technologies - Molecular Probes	A21206
		Alexa Fluor 568-goat- anti-Chicken IgG (H+L)	Life Technologies - Molecular Probes	A11041
		Alexa Fluor Cy3 Donkey anti-Goat IgG (H+L)	Jackson ImmunoResearch	705-165-147
		Alexa Fluor Cy3 Donkey anti-Mouse IgG (H+L)	Jackson ImmunoResearch	715-165-151
		Alexa Fluor Cy3 Donkey anti-Rat IgG (H+L)	Jackson ImmunoResearch	712-165-150
		Alexa Fluor Cy3 Donkey anti-Rabbit IgG (H+L)	Jackson ImmunoResearch	711-165-152
		Alexa Fluor 647 Donkey anti-Goat IgG (H+L)	Life Technologies - Molecular Probes	A21447
		Alexa Fluor 647 (ab')$_2$ fragment Donkey anti-Mouse IgG (H+L)	Jackson ImmunoResearch	715-606-150

		Alexa Fluor 647-donkey anti-Rat IgG (H+L)	Jackson ImmunoResearch	712-605-153
		Alexa Fluor 647-donkey anti-Rabbit IgG (H+L)	Jackson ImmunoResearch	711-605-152
	Nucleic Acid Stain	SYTO16	Life Technologies - Molecular Probes	S7578
		Hoechst33342	Life Technologies - Molecular Probes	H3570
**Imaging**	RIMS	Histodenz	Sigma-Aldrich	D2158
	CUBIC-mount	Sucrose	Junsei chemical	31365-0301
		Urea	Affymetrix	23036
		N,N,N’,N’-tetrakis(2-hydroxypropyl)ethylenediamine	Sigma-Aldrich	122262
	Dish	Coverglass bottom dish	SPL lifesciences	101350

Supplementary Table 2. Antibodies tested on Active Clarity Technique (ACT)-processed tissues

**NO.**	**Antibody**	**Species**		**Antigen localization**	**Company**	**Cat #**	**Dilution**
**1**	**Acetylated Tubulin**	Mouse	Monoclonal	Cytoplasm	Sigma	T7451	1:500
**2**	**Active caspase3**	Rabbit	Polyclonal	Cytoplasm	Cell Signaling	9661	1:500
**3**	**Bcl-2**	Mouse	Monoclonal	Cytoplasm/ Mitochondria	Santa Cruz Biotech	Sc7382	1:250
**4**	**β-catenin**	Mouse	Monoclonal	Cytoplasm	BD Biosciences	610153	1:500
**5**	**β-Tubulin III (Tuj1)**	Mouse	Monoclonal	Cytoplasm	Covance	MMS-435P	1:350
**6**	**β-Tubulin III (Tuj1)**	Mouse	Monoclonal	Cytoplasm	Sigma	T8660	1:300
**7**	**β-Tubulin III (Tuj1)**	Rabbit	Polyclonal	Cytoplasm	Sigma	T2200	1:300
**8**	**Brain lipid-binding protein (BLBP)**	Rabbit	Polyclonal	Cytoplasm	Chemicon	AB9558	1:500
**9**	**Bromodeoxyuridine (BrdU)**	Rat	Monoclonal	Nuclei	Abcam	ab6326	1:500

**10**	**Calbindin D-28k**	Mouse	Monoclonal	Cytoplasm	Swant	300	1:500
**11**	**Calbindin D-28k**	Rabbit	Polyclonal	Cytoplasm	Millipore	AB1778	1:500
**12**	**Calretinin**	Rabbit	Polyclonal	Cytoplasm	Swant	7697	1:500
**13**	**Calretinin**	Rabbit	Polyclonal	Cytoplasm	Millipore	AB5054	1:800
**14**	**Caveolin**	Rabbit	Polyclonal	Membrane	Abcam	ab18199	1:300
**15**	**CD133**	Rat	Monoclonal	Membrane	Millipore	MAB4310	1:500
**16**	**CD31**	Rat	Monoclonal	Membrane	BD Biosciences	557355	1:500
**17**	**c-Fos**	Rabbit	Polyclonal	Nuclei	Santa Cruz Biotech	SC253	1:500
**18**	**Calcitonin gene-related peptide (CGRP)**	Goat	Polyclonal	Cytoplasm	Abcam	ab36001	1:300
**19**	**Choline acetyltransferase (ChAT)**	Goat	Polyclonal	Cytoplasm	Millipore	AB144P	1:100
**20**	**2',3'-cyclic nucleotide 3'-phosphodiesterase (CNPase)**	Mouse	Monoclonal	Membrane	Millipore	MAB326	1:300
**21**	**2',3'-cyclic nucleotide 3'-phosphodiesterase (CNPase)**	Mouse	Monoclonal	Membrane	Sigma	c5922	1:300
**22**	**Collagen type III**	Rabbit	Polyclonal	Extracellular matrix	Abcam	AB7778	1:300
**23**	**Collagen type Ⅳ**	Rabbit	Polyclonal	Extracellular matrix	Abcam	AB6586	1:300
**24**	**Ctip2**	Rat	Monoclonal	Nuclei	Abcam	ab18465	1:800
**25**	**Cullin 4 (CUL4)**	Rabbit	Polyclonal	Cytoplasm	Santa Cruz Biotech	sc-13024	1:150
**26**	**Cut-like homeobox 1 (Cux1)**	Rabbit	Polyclonal	Nuclei	Santa Cruz Biotech	sc-13024	1:150
**27**	**Doublecortin (DCX)**	Goat	Polyclonal	Cytoplasm	Santa Cruz Biotech	sc-8066	1:500
**28**	**Epidermal growth factor receptor (EGFR)**	Rabbit	Polyclonal	Membrane	Abcam	ab2430	1:500
**29**	**Gamma-aminobutyric acid (GABA)**	Rabbit	Polyclonal	Secretion	Sigma	A2052	1:500

**30**	**Glutamic acid decarboxylase 67 (GAD67)**	Mouse	monoclonal	Cytoplasm	Millipore	MAB5406	1:500
**31**	**Growth associated protein 43 (GAP43)**	Rabbit	Polyclonal	Cytoplasm	Abcam	ab16053	1:500
**32**	**Glial fibrillary acidic protein (GFAP)**	Mouse	monoclonal	Cytoplasm	Cell Signaling	#3670	1:500
**33**	**Glial fibrillary acidic protein (GFAP)**	Rabbit	Polyclonal	Cytoplasm	Abcam	ab7260	1:800
**34**	**Glial fibrillary acidic protein (GFAP)**	Rabbit	Polyclonal	Cytoplasm	Dako	Z0334	1:500
**35**	**Glial fibrillary acidic protein (GFAP)**	Rat	Monoclonal	Cytoplasm	Invitrogen	130330	1:500
**36**	**Green fluorescent protein (GFP)**	Chicken	Polyclonal	-	Abcam	AB13970	1:500
**37**	**Green fluorescent protein (GFP)**	Rabbit	Polyclonal	-	Abcam	AB290	1:300
**38**	**Glucose transporter 1 (GLUT1)**	Rabbit	Polyclonal	Membrane	Abcam	ab15309	1:800
**39**	**Glucose transporter 1 (GLUT1)**	Rabbit	Polyclonal	Membrane	Thermo	PA1-21041	1:300
**40**	**Heme oxygenase1 (HO-1)**	Mouse	Monoclonal	Endoplasmic reticulum	Abcam	AB13248	1:250
**41**	**Homeobox only protein homeobox (HOPX)**	Rabbit	Polyclonal	Nuclei	Sigma	hpa030180	1:500
**42**	**Ionized calcium binding adapter molecule 1 (Iba1)**	Rabbit	Polyclonal	Cytoplasm	Waco	019-19741	1:500
**43**	**Inositol 1,4,5-trisphosphate 3-kinase A (IP3KA)**	Goat	Polyclonal	Cytoplasm	Santa Cruz Biotech	sc11206	1:500
**44**	**Laminin**	Rabbit	Polyclonal	Extracellular matrix	Sigma	L9393	1:500
**45**	**Microtubule-associated protein 2 (MAP2)**	Mouse	Monoclonal	Cytoplasm	Millipore	MAB3418	1:500
**46**	**Microtubule-associated protein 2 (MAP2)**	Rabbit	Polyclonal	Cytoplasm	Millipore	AB5622	1:500

**47**	**Microtubule-associated protein 2 (MAP2)**	Rabbit	Polyclonal	Cytoplasm	Cell Signaling	4542	1:350
**48**	**Microtubule-associated protein 2B (MAP2B)**	Mouse	Monoclonal	Cytoplasm	BD Biosciences	610460	1:300
**49**	**Mammalian achaete scute homolog-1 (Mash1)**	Mouse	Monoclonal	Nuclei	BD Biosciences	556604	1:500
**50**	**Myelin basic protein (MBP)**	Chicken	Polyclonal	Membrane	Aves lab	mbp	1:300
**51**	**Myelin basic protein (MBP)**	Rabbit	Polyclonal	Membrane	Abcam	ab40390	1:500
**52**	**Neural cell adhesion molecule (NCAM)**	Rabbit	Monoclonal	Membrane	Millipore	AB5032	1:250
**53**	**Nestin**	Mouse	Monoclonal	Cytoplasm	Millipore	MAB353	1:500
**54**	**Neuronal nuclear antigen (NeuN)**	Mouse	Monoclonal	Nuclei	Millipore	MAB377	1:500
**55**	**Neurofilament-H**	Mouse	Monoclonal	Cytoplasm	Cell Signaling	2836	1:300
**56**	**Neurofilament H Non-Phosphorylated (SMI32)**	Mouse	Monoclonal	Cytoplasm	Covance	SMI-32R	1:500
**57**	**Neurofilament-M (NFM)**	Mouse	Monoclonal	Cytoplasm	Santa Cruz Biotech	sc-51683	1:500
**58**	**Neuron-glial antigen 2 (NG2)**	Rabbit	Polyclonal	Membrane	Millipore	AB5320	1:500
**59**	**Nitric oxide synthase 1 (NOS1)**	Rabbit	Polyclonal	Membrane	Santa Cruz Biotech	sc-648	1:250
**60**	**Oligodendrocyte Marker O4 (O4)**	Mouse	Monoclonal	Membrane	Millipore	MAB345	1:250
**61**	**Oligodendrocyte lineage transcription factor 2 (Olig2)**	Rabbit	Polyclonal	Nuclei	Millipore	AB9610	1:300

**62**	**Oligodendrocyte lineage transcription factor 2 (Olig2)**	Rabbit	Polyclonal	Nuclei	IBL	JP-18953	1:250
**63**	**Olfactory marker protein (OMP)**	Rabbit	Polyclonal	Cytoplasm	Thermo	OSP0000 1W	1:250
**64**	**Parvalbumin**	Mouse	Monoclonal	Cytoplasm	Millipore	MAB1572	1:500
**65**	**Proliferating cell nuclear antigen (PCNA)**	Mouse	Monoclonal	Nuclei	Santa Cruz Biotech	Sc-56	1:250
**66**	**Platelet-derived growth factor receptors (PDGFR)**	Rat	Monoclonal	Membrane	BD Biosciences	558774	1:500
**67**	**Protein gene product 9.5 (PGP9.5)**	Rabbit	Polyclonal	Cytoplasm	Upstate Biotechnology	06-570	1:500
**68**	**Polysialylated neuronal cell adhesion molecule (PSA-NCAM)**	Mouse	Monoclonal	Membrane	Millipore	MAB5324	1:500
**69**	**Postsynaptic density protein 95 (PSD95)**	Mouse	Monoclonal	Membrane	Thermo	6G6-1C9	1:500
**70**	**Postsynaptic density protein 95 (PSD95)**	Rabbit	Polyclonal	Membrane	Invitrogen	51-6900	1:500
**71**	**Regulated in development and DNA damage responses 1 (REDD1)**	Rabbit	Polyclonal	Membrane	ptglab	10638-1-AP	1:250
**72**	**RFP**	Rabbit	Polyclonal	-	Abcam	AB62341	1:300
**73**	**s100**	Mouse	Monoclonal	Membrane	Sigma	s2532	1:500
**74**	**Special AT-rich sequence-binding protein 2 (SATB2)**	Rabbit	Monoclonal	Nuclei	Abcam	ab34735	1:500
**75**	**Smooth muscle protein 22-alpha (SM22 alpha)**	Rabbit	Polyclonal	Membrane	Abcam	ab14106	1:300
**76**	**Sox2**	Rabbit	Polyclonal	Nuclei	Millipore	AB5603	1:500

**77**	**Tropomyosin receptor kinase A (TrkA)**	Goat	Polyclonal	Membrane	R&D systems	AF1056	1:500
**78**	**Trombosphondin 4**	Goat	Polyclonal	Cytoplasm	R&D systems	AF3074	1:500
**79**	**Tyrosine hydroxylase (TH)**	Rabbit	Polyclonal	Cytoplasm	Millipore	AB152	1:300
**80**	**Tyrosine hydroxylase (TH)**	Mouse	Monoclonal	Cytoplasm	Santa Cruz Biotech	sc14007	1:250
**81**	**Vesicular glutamate transporter 1 (VGlut1)**	Mouse	Monoclonal	Membrane	Synaptic Systems	135311	1:500
**82**	**Vimentin**	Goat	Polyclonal	Cytoplasm	Millipore	AB1620	1:300

Supplementary Table 3. Electrophoretic tissue clearing (ETC) and PRESTO material information

	Product	company	Cat #
ETC chamber	ECT chamber	Logos Biosystems, Inc.	C10101
	ECT chamber controller	Logos Biosystems, Inc.	C10201
	Temperature probe	Logos Biosystems, Inc.	C12101
	Peristatic pump	Baoding longer precision pump Co., Ltd	YZ1515X
	Buffer reservoir	Logos Biosystems, Inc.	C10401
	Tissue container	Logos Biosystems, Inc.	C12001
	Container holder for 1 tissue container	Logos Biosystems, Inc.	C12002
	Mouse brain slice holder	Logos Biosystems, Inc.	C12004
	Whole rat brain holder	Logos Biosystems, Inc.	C12007
	Peristaltic pump tubing	Logos Biosystems, Inc.	C12104
c-PRESTO	Tabletop-centrifuge	Hanil science industrial Co., Ltd	MICRO 12
	Micro tube 1.5ml	SARSTEDT	D-51588
s-PRESTO	Syringe pump	Baoding longer precision pump Co., Ltd	LSP02-1B
	Syringe (20 ml)	Korea vaccine Co., Ltd.	KV-S20
	3-way stopcock	Hyupsung medical Co.,Ltd.	HS-T-01N

Supplementary Table 4. Electrophoretic tissue clearing (ETC) conditions tested tissues

Tissues			Optimized ETC time (hour)	Constant current	ETC Clearing solution	Clearing solution volume / Replacement interval of clearing solution
Mouse	brain	1mm slice	1 - 2	1.5 A	DW based	1 L / no need
		2-3mm slice	3 - 4	1.5 A	DW based	1 L / no need
		whole	5 - 6	1.5 A	DW based	1 L / no need
	Body		24	1.5 A	DW based	1.5 L / 10-12 hours
	Intestine		2 – 3	1.5 A	DW based	1 L / no need
	Lung		4 - 5	1.5 A	DW based	1 L / no need
	Liver		20	1.5 A	0.01 M PBS based	1.2 L / 10-12 hours
	Kidney		20	1.5 A	DW based	1.2 L / 10-12 hours
	Spleen		20	1.5 A	0.01 M PBS based	1.2 L / 10-12 hours
	Testis		4 – 5	1.5 A	0.01 M PBS based	1 L / no need
	Thymus		5	1.5 A	DW based	1 L / no need
Rat	Embryo		9 – 10	1.5 A	0.01 M PBS based	1.2 L / no need
	Brain (Hemisphere)		8	1.5 A	DW based	1 L / no need
Rabbit brain (Hemisphere)			50	1.5 A	DW based	1.2 L / 10-12 hours
Chicken			6 - 12	1.5 A	0.01 M PBS based	1.2 L / no need
Frog			24	1.5 A	DW based	1.2 L / 10-12 hours
Zebrafish			4 - 6	1.5 A	0.01 M PBS based	1 L / no need
Human brain slice			72	1.5 A	DW based	1.2 L /10 -12 hours
Human spinal cord			100	1.5 A	DW based	1 L / 10 -12 hours

Supplementary Video 1. Real-time progress of whole brain clearing using the Active Clarity Technique (ACT) shown in Fig. 1

Supplementary Video 2. Three-dimensional visualization of the motoneurons in the intact mouse spinal cord (8-weeks-old) shown in Supplementary Fig. 1

A series of images from the ventral surface to the center of the intact mouse spinal cord shown in Supplementary Fig. 1. The video shows immunolabeled SMI32-positive motoneurons (stack size, 1,188 μm; step size, 2 μm).

Supplementary Video 3. Three-dimensional visualization of a 1-mm thickness coronal block of the adeno-associated virus-green fluorescent protein (AAV-GFP) injected mouse brain (8-weeks-old) shown in Fig. 2

A series of images showing that immunolabeled dendritic arbors can be clearly identified in the AAV-GFP injected mouse brain (stack size, 1,140 μm; step size, 2 μm).

ACT-PRESTO PROTOCOL

Eunsoo Lee[1], Jungyoon Choi[1], Youhwa Jo[1], Joo Yeon Kim[1], Yu Jin Jang[2], Hye Myeong Lee[2], So Yeun Kim[3], Ho-Jae Lee[4], Keunchang Cho[4], Neoncheol Jung[4], Eun Mi Hur[5,6], Sung Jin Jeong[2], Cheil Moon[3], Youngshik Choe[2], Im Joo Rhyu[1], Hyun Kim[1], and Woong Sun[1]

[1]Department of Anatomy and Division of Brain Korea 21 Plus Program for Biomedical Science, Korea University College of Medicine, Anam-dong, Seongbuk-gu, Seoul 136-705, Korea; [2]Department of Neural Development and Disease, Korea Brain Research Institute, 701-300 Daegu, Korea; [3]Department of Brain & Cognitive Sciences, Graduate School, Daegu Gyeungbuk Institute of Science and Technology (DGIST), Daegu, Korea; [4]Logos Biosystems, Inc. Anyang-Si, Gyunggi-Do, 431-755, Republic of Korea; [5]Center for Neuroscience, Brain Science Institute, Korea Institute of Science and Technology, Seoul, Korea, [6]Department of Neuroscience, Korea University of Science and Technology (UST), Daejeon, Korea

ACT: mouse whole-brain clearing

Sample preparation & fixation:

1. Sacrifice mice with an overdose of anesthesia.
2. Transcardially perfuse with 0.9% NaCl (pH 7.4–7.5) containing heparin and then with 4% PFA in 0.1 M PBS (pH 7.4).
3. Post-fix the excised whole mouse brain with 4% PFA in 0.1 M PBS at 4°C overnight.

   Note: Longer fixation could elongate clearing time. Optimal fixation time should be determined empirically. A 2-hour post-fixation is usually sufficient for 1–2 mm brain slices.

Hydrogel monomer infusion & polymerization

1. Prepare the A4P0 hydrogel monomer solution.

   Take 40 ml of 4% acrylamide in 0.1 M PBS solution and add 100 mg of VA-044 Initiator to make a final concentration of 0.25%.
2. Whole brain or sliced sections are incubated at 4°C for 12–24 hours in A4P0 hydrogel monomer solution.

3. De-gas the hydrogel-infused samples submerged in the hydrogel monomer by bubbling nitrogen for 3 min through a 5 ml of sample-hydrogel solution in a 10 ml tube.

4. Transfer the samples to a water-bath (37°C) for 2 hours.

   Note: Although tissue-infused hydrogels gel by cross-linking with endogenous macromolecules, the free A4P0 solution is not polymerized. Therefore, no gel is expected to form outside of the tissues.

5. Wash the polymerized samples briefly with 0.01 M PBS to remove excess hydrogel.

Electrophoretic tissue clearing (ETC)

1. Transfer samples into the tissue container (see Fig. **a** below) of the ETC chamber and place the tissue container in the ETC chamber (see Fig. **b** below). The ETC chamber is filled with ETC running buffer (clearing solution).

   Note: ETC running buffer (clearing solution): 4% SDS and 200 mM boric acid in dH_2O (pH 8.5)

2. Set the ETC conditions and run the ETC for 6 hours (see Fig. **c** below and **Supplementary Table 2**).

   Note: ETC settings: current (1.5Å), temperature (37°C), switching time (0)

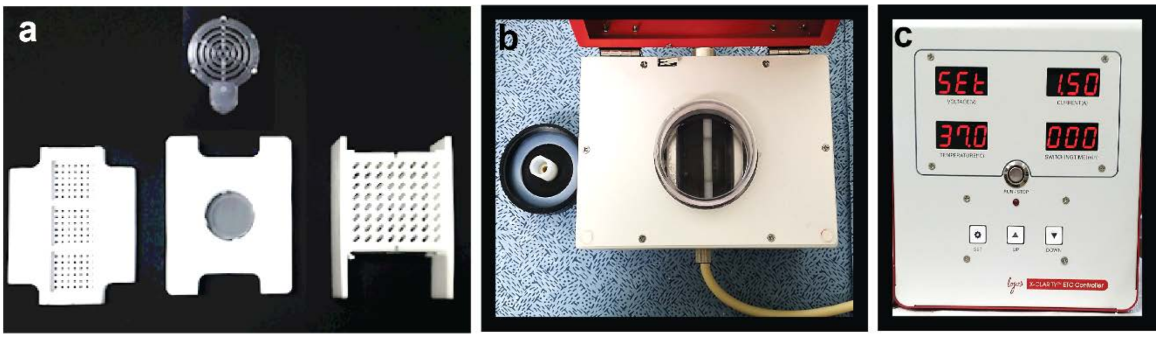

a. Tissue container and holder; mouse brain slice holder, container for one brain, and container for large organs (from *left*). **b.** ETC chamber. The container is placed inside the ETC chamber. **c.** Set the ETC conditions of 1.5Å, 37°C, and zero switching time.

3. Wash the cleared samples with 0.01 M PBS and change buffers occasionally until no

SDS bubbles are seen after brief shaking of the cleared tissue.

Immunolabeling ACT-processed brain tissue

Incubate whole-brain samples for 4 days in primary antibody diluted in blocking buffer at 37°C with mild shaking and replace the antibody solutions on day 2. An overnight incubation at 37°C is sufficient for 1–2 mm cut samples.

1. Wash samples in 0.1 M PBS for 3–5 hours and change the buffer every hour.
2. Shake incubate samples in secondary antibody diluted in blocking buffer for 4 days at 37°C.
3. Wash samples in 0.1 M PBS for 3–5 hours, and change buffer every hour.
4. Transfer samples to a refractive index matching media (RIMS, CUBIC-mount, or Focus clear) for imaging.

   Note: Donkey serum can be used instead of BSA in cases of high background.

PRESTO immunolabeling with dense tissues

c-PRESTO

c-PRESTO is suitable for small-sized samples, such as whole testis, kidney, or small parts of larger organs.

1. Transfer samples into an e-tube, add 400 µl of the primary antibody solution (1:100–500, in 0.1 M PBS containing 6% BSA and 0.1% Triton X-100), and centrifuge the e-tube at 600 × g for 2 hours.
2. Wash the stained sample with 0.01 M PBS by centrifugation at 600 × g for 30 min.
3. Add secondary antibody solution (1:100–500 dilution in 0.1 M PBS containing 6% BSA and 0.1% Triton X-100) and centrifuge at 600 × g for 2 hours.
4. Repeat step 2.

s-PRESTO

s-PRESTO is suitable for larger tissues.

1. Prepare the syringe and connect a 3-way valve to the syringe (see Figs. **a** and **b** below).
2. Glue the valve with a glue gun for the high pressure conditions (see Fig. **b** below).
3. Set the syringe pump conditions. Infusion/withdrawal volume at 10 ml/min and a 4 min pause time on continuous cycle mode (see Figs **c** and **d** below).

   Note: The pump infuses until reaching the target volume (10 ml) and then the direction of flow changes after a brief pause (4 min).
4. Transfer sample and primary antibody solution (2–5 ml of 1:500–800 in 0.1 M PBS containing 6% BSA and 0.1% Triton X-100) into the syringe (see Fig. **e** below). This can be done in the “open” valve position.
5. Close the valve with the piston at the 15-ml position to provide sufficient room for the infusion/withdrawal movement of the piston.
6. Set the syringe on the pump.
7. Run the syringe pump for 3–24 hours at room temperature (see Fig. **f** below).
8. Open the valve and replace the solution with 0.01 M PBS.
9. Wash the stained sample twice with 0.01 M PBS each 1 hour using the syringe pump.
10. Add secondary antibody solution (1:100–500 dilution in 0.1 M PBS containing 6% BSA and 0.1% Triton X-100) and run the syringe pump for 3–24 hours.
11. Repeat steps 8–9.

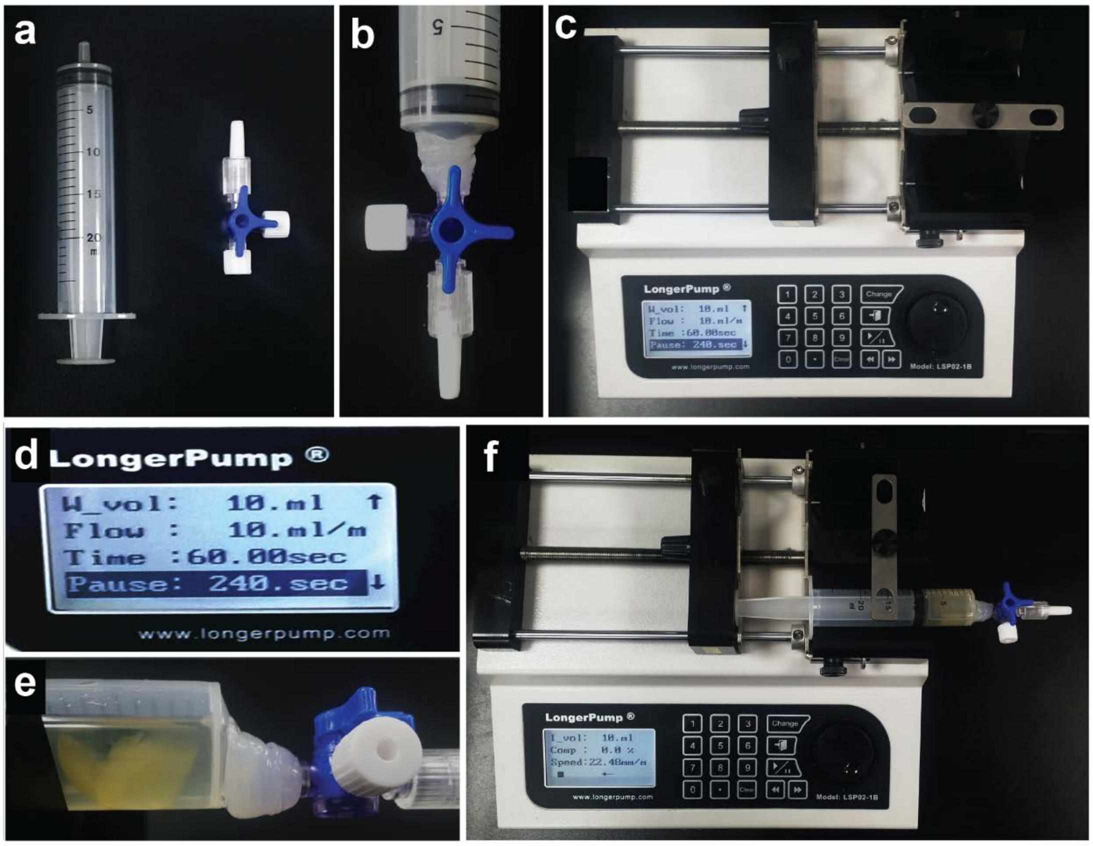

a. Syringe (20 ml) and three-way stopcock. **b.** Three-way stopcock connected and glued to the syringe. **c, d.** Syringe pump and working condition setup. **e.** Syringe containing sample and antibody solution. Note that sufficient space is required for piston movement. **f.** Syringe placed on the syringe pump.
